# An efficient antimicrobial depot for infectious site-targeted chemo-photothermal therapy

**DOI:** 10.1186/s12951-018-0348-z

**Published:** 2018-03-16

**Authors:** Menglong Liu, Danfeng He, Tao Yang, Wei Liu, Li Mao, Yang Zhu, Jun Wu, Gaoxing Luo, Jun Deng

**Affiliations:** 1 Institute of Burn Research, Southwest Hospital, State Key Lab of Trauma, Burn and Combined Injury, Third Military Medical University (Army Medical University), Chongqing, 400038 China; 2 Department of Laboratory Medicine, Southwest Hospital, Third Military Medical University, (Army Medical University), Chongqing, 400038 People’s Republic of China; 3 Department of Burns, The First Affiliated Hospital, SunYat-Sen University, Guangzhou, 510080 China; 4 Departments of Bioengineering and Materials Science and Engineering, University of California, Berkeley, CA 94720 USA

**Keywords:** Drug-resistant bacteria, Combinational chemo-hyperthermia therapy, Charge conversion, pH-sensitive release, Bacterium-specific targeting

## Abstract

**Background:**

Silver and photothermal therapy (PTT) have been widely used for eradicating the drug-resistant bacteria. However, the risks of excess of silver for humans and the low efficiency of PTT still limit their in vivo therapeutic application. Integration of two distinctive bactericides into one entity is a promising platform to improve the efficiency of antimicrobial agents.

**Results:**

In this study, a chemo-photothermal therapeutic platform based on polydopamine (PDA)-coated gold nanorods (GNRs) was developed. The PDA coating acquired high Ag^+^ ions loading efficiency and Cy5-SE fluorescent agent labeled glycol chitosan (GCS) conjugation (Ag^+^-GCS-PDA@GNRs). This platform became positively charged in the low pH environment of the abscess, allowing their accumulation in local infection site as revealed by thermal/florescence imaging. The loaded Ag^+^ ions was released in a pH-sensitive manner, resulting in selective Ag^+^ ions delivery to the abscess environment (pH ~ 6.3). More importantly, the ultralow dose of Ag^+^ ions could effectively damage the bacterial membrane, causing the permeability increase and the heat resistance reduction of the cell membrane, leading to the large improvement on bactericidal efficiency of PTT. On the other hand, the hyperthermia could trigger more Ag^+^ ions release, resulting in further improvement on bactericidal efficiency of chemotherapy. Combinational chemo-hyperthermia therapy of Ag^+^-GCS-PDA@GNRs could thoroughly ablate abscess and accelerate wound healing via a synergistic antibacterial effect.

**Conclusions:**

Our studies demonstrate that Ag^+^-GCS-PDA@GNRs is a robust and practical platform for use in chemo-thermal focal infection therapy with outstanding synergistic bacteria ablating.

**Electronic supplementary material:**

The online version of this article (10.1186/s12951-018-0348-z) contains supplementary material, which is available to authorized users.

## Background

Infectious diseases caused by bacteria, especially the drug-resistant bacteria have become a major and even fatal global human health issue [1, 2]. According to a recent report, failure in management of drug-resistant infections may cause more than 10 million deaths per year and cost up to 100 trillion dollars by 2050 [3, 4]. Silver nanoparticles (AgNPs) exhibited broad-spectrum antimicrobial activity with no microbial resistance [5, 6]. However, excess AgNPs could cause argyria, leading to spasms, gastrointestinal disorders, and even deaths [5, 7]. This limited the scope of therapeutic applications of AgNPs-based antibacterial agents. Currently, AgNPs are considered to disturb essential bacterial cell functions through two dominating mechanisms [8]. In the first mechanism, silver ions (Ag^+^) released from the AgNPs interact with the proteins and enzymes, resulting in a serious structural deformation of the bacterial cell membrane. The second mechanism involves the production of high concentration of reactive oxygen species (ROS), which perturbs the cell metabolism [5, 6]. Although the exact antimicrobial mechanism of the AgNPs is still in debate, it is certain that the local effect of silver ions released from the core of AgNPs at the cellular walls of both bacteria and human cells contributes to their efficacy as well as toxicity [7, 9, 10]. Therefore, strategies that minimize Ag^+^ ions concentration (instead of entire AgNPs core) while maintaining high antibacterial efficiency are needed. Although efforts have been made in this direction [8, 11], it is still difficult to lower the concentration of Ag^+^ ions without sacrificing antibacterial efficiency. In addition to the toxicity issue, Ag^+^ ions release or migration in undesired locations increases the risk of associated complications.

Laser irradiation-assisted photothermal therapy (PTT) converts near-infrared (NIR, 700–1100 nm) light energy into heat, which rapidly kills bacterial cells by rising local temperature [12–15]. PTT is attractive as laser can be applied noninvasively and focused onto desired locations. So far, the bactericidal efficiency of PTT is still relatively low in spite of many efforts that have been devoted to improve corresponding outcome of local infection treatment [14, 16]. On the other hand, current working temperatures of PTT are too high (55–60 °C), which can potentially burn skin and damage other normal tissues [14, 15]. Combination therapy is a widely adopted concept in bacterial treatment, which achieves improved therapeutic efficiency by combining two distinct fronts compared to sole usage of each [6, 17]. Inspired by this, we hypothesize that combination therapy of hyperthermia and silver ions could make up for the deficiencies of the single modal antibacterial process and show synergistic antibacterial activities.

On the other hand, anti-bacterial agents are administrated locally in most studies about treatments of bacteria-associated infections [6, 8, 11, 18–20]. However, local administration is not suitable for infection sites that are deep or widespread in the body. Systemic administration of anti-bacterial agents that can target to infection sites could theoretically overcome these drawbacks. Acidity within bacterial infection sites attributed to a combination of low oxygen tension triggering anaerobic fermentation [21, 22] and the production of organic acids has been demonstrated for highly diverse bacteria strains including methicillin-resistant *Staphylococcus aureus* (MRSA) [23, 24]. Therefore, integration of photothermal and silver ions therapeutic agents on a combined platform incorporated with acidity responsiveness is of great promise.

**Scheme 1:**
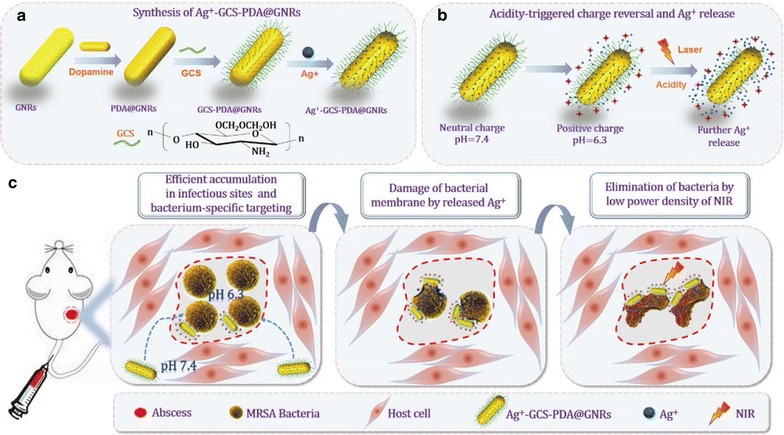
Schematic of (**a**) synthesis of Ag^+^-GCS-PDA@GNRs, (**b**) acidity-triggered charge reversal and Ag^+^ ions release, and (**c**) bacteria-specific targeting and chemo-photothermal combinational therapy based on the Ag^+^-GCS-PDA@GNRs

In this study, we developed a bacterium-targeted therapeutic platform based on the polydopamine (PDA)-coated gold nanorods (PDA@GNRs), which combined the chemo-photothermal therapy together. Firstly, we coated GNRs with PDA, and then modified with glycol chitosan (GCS), a water-soluble chitosan derivative with a pH-variant charge (pK_a_ ~ 6.5) and stealth properties [14]. Finally, Ag^+^ ions were loaded on the GCS-PDA@GNRs to form an efficient and benign antimicrobial depot (Ag^+^-GCS-PDA@GNRs) (Scheme 1a). In addition, the free amines on the GCS were employed to graft imaging agents (Cy5SE). We hypothesize that the antimicrobial depot will be able to circulate freely in the bloodstream and accumulate in acidic infectious site where they become positively charged, and selectively target themselves to negatively charged bacterial cell surfaces (Scheme 1b). With the external light-activation, they will generate heat in the close vicinity of the target bacteria (Scheme 1c). The acidity/thermal-triggered Ag^+^ ions release may reduce the heat resistance of the bacteria and PTT may promote the antibacterial efficiency of the Ag^+^ ions. It is expected that the innovative antimicrobial depot could greatly reduce the dosage of silver and show enhanced bactericidal efficiency as well as excellent biocompatibility. The hypothesized targeting feature and synergistic effect of chemo-photothermal therapy of Ag^+^-GCS-PDA@GNRs will be examined with a murine model against MRSA infectious abscesses (pH 6.3).

## Results and discussion

### Characterizations

**Fig. 1:**
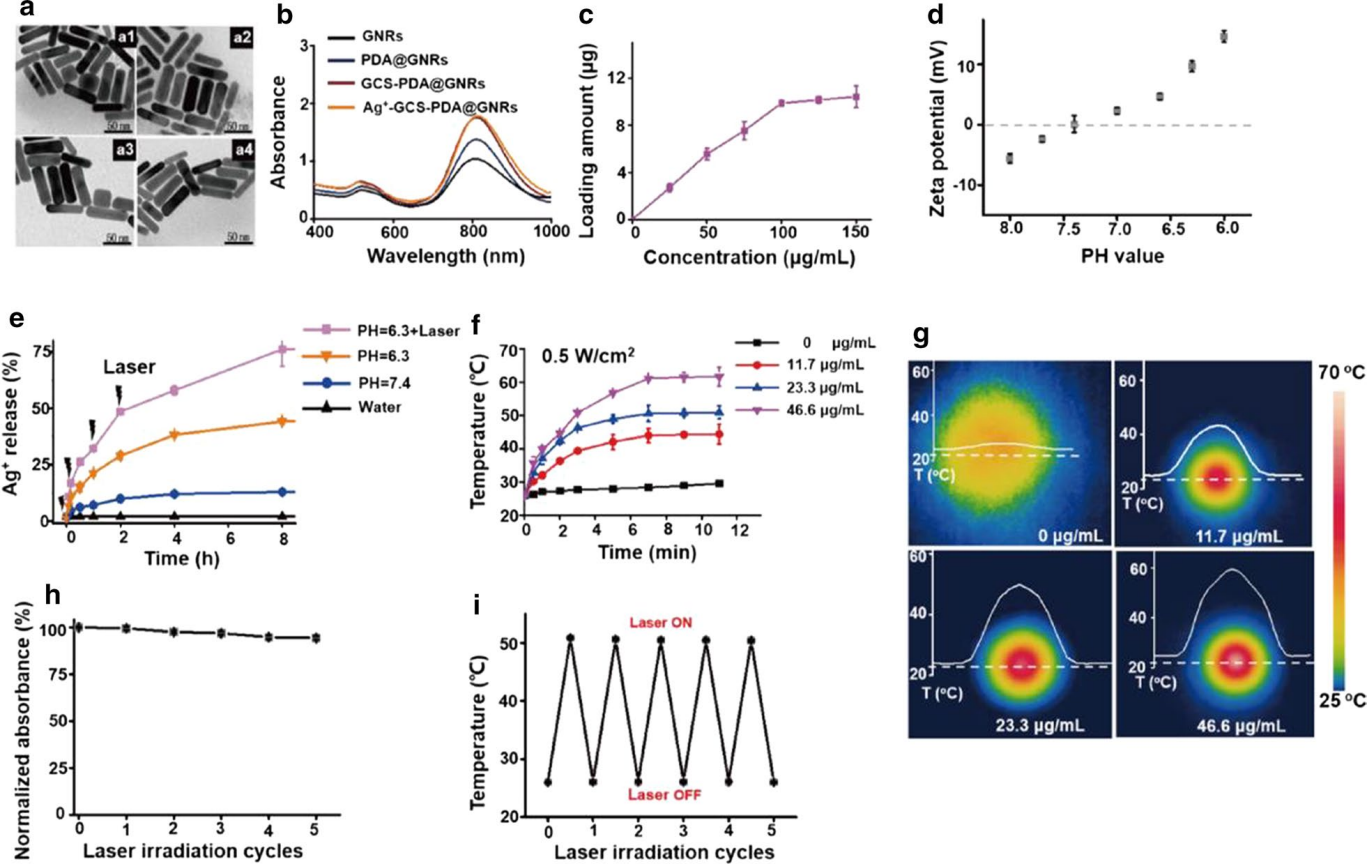
Characterization of Ag^+^-GCS-PDA@GNRs. **a** Representative TEM images of (a1) GNRs, (a2) PDA@GNRs, (a3) GCS-PDA@GNRs, and (a4) Ag^+^-GCS-PDA@GNRs. **b** UV–vis-NIR spectra of GNRs, PDA@GNRs, GCS-PDA@GNRs and Ag^+^-GCS-PDA@GNRs, respectively. **c** The zeta potential of Ag^+^-GCS-PDA@GNRs in PB with different pH values (7.4 & 6.3). **d** Quantitative analysis of Ag^+^ ions loading capacity of PDA@GNRs. **e** The release curve of Ag^+^ ions from Ag^+^-GCS-PDA@GNRs in medium at pH 7.4 and 6.3 with or without laser irradiation for a certain time, respectively. **f** Temperature evolution profile of PB buffer and Ag^+^-GCS-PDA@GNRs suspensions with different concentrations in PB (pH 6.3) upon NIR laser (808 nm, 0.5 W cm^−2^) irradiation. **g** The corresponding thermographic images of different concentrations of Ag^+^-GCS-PDA@GNRs suspension in PB (pH 6.3) under the NIR irradiation. **h** The change in absorbance intensity of Ag^+^-GCS-PDA@GNRs after repeated laser irradiation (n = 5; 808 nm, 0.5 W cm^−2^). **i** Change in thermal curves of Ag^+^-GCS-PDA@GNRs after repeated laser irradiation (n = 5; 808 nm, 0.5 W cm^−2^)

The gold nanorods (GNRs) were synthesized by seed growth approach using a surfactant of CTAB [25–27]. FTIR spectrum analysis of GNRs after purification revealed that most of the feature peaks were attributed to the CTAB, including the CH_2_ scissor bending vibration at 1398 cm^−1^, and the CH_2_ symmetric and anti-symmetric telescoping vibrations at 2842 and 2926 cm^−1^, and the anti-symmetric telescoping vibration for the head group of N^+^ at 1472 cm^−1^, as well as the C^−^ and N^+^ telescoping vibration at 911 and 1153 cm^−1^, respectively [28] (Additional file 1: Figure S1A). Meanwhile, the characteristic peaks at 683 and 2339 cm^−1^ were from the CO_2_ flexural and telescoping vibrations, separately. Transmission electron microscopy (TEM) analysis exhibited that GNRs had an uniform morphology and size with an average length of 68 ± 2 nm and diameter of 21 ± 1 nm [aspect ratio of ~ 3.3; Fig. 1a (a1)]. The local surface plasmon resonance (LSPR) peak of GNRs was evaluated by UV–vis-NIR spectroscopy, and the longitudinal and transverse LSPR peaks at about 800 nm and 510 nm were observed from the UV–Vis-NIR spectra of GNRs, respectively (Fig. 1b). The surface charge of CTAB protected GNRs was measured by zeta potential measurement, and its zeta potential was 40.1 mV, which was considered to be a result of the presence of a bilayer of CTAB on GNRs surface (Additional file 1: Figure S2A) [29].

Dopamine had excellent performance of self-polymerization and could spontaneously deposit on GNRs surface forming the polydopamine (PDA) coating under alkaline conditions (pH, 8.5) [30]. In addition, the PDA coating also could suppress the cytotoxicity of CTAB [29] as well as allow the further modification of functionalized ions, molecules or polymers due to its unique structure [31]. Following synthesis and purification, the structure of PDA and PDA@GNRs was analyzed by FTIR (Additional file 1: Figure S1A). The FTIR spectrum of PDA revealed that the peaks located around 1510, 1610 and 2937 cm^−1^ were attributed to the C-H frame vibration and stretching vibration in the aromatic ring, respectively [32, 33]. Meanwhile, the broad band appeared among 3200–3600 cm^−1^ was associated with the telescoping vibration of -OH groups [32, 33]. The FTIR spectrum of PDA@GNRs detected that most of the feature peaks of the CTAB were replaced by the characteristic peaks of PDA coating, which gave a credible proof of successful synthesis of PDA@GNRs. As shown in Fig. 1a (a2), the high-magnification TEM micrograph of PDA protected GNRs (PDA@GNRs) showed that almost no morphological and size change occurred after PDA coating, which illustrated that the coating uniformly coated onto GNRs and the thickness of PDA coating was fairly thin. Compared with the LSPR peak of GNRs, the transverse LSPR peak of the PDA@GNRs had no obvious change but its transverse LSPR peak showed a slight red-shift phenomenon (~ 10 nm; Fig. 1b). It’s worth noting that there was a noticeable improvement in longitudinal plasmon band maxima under the accordant concentration of GNRs because the structure of PDA contained auxochrome of hydroxyl groups, which could increase the activity range of the electrons [34, 35]. Hence, this provided another strong evidence of the successful deposition of PDA coating on GNRs. The zeta potential of GNRs showed a decrease (28.5 mV) after the PDA coating, and this decline was probably due to the hydroxyl groups in PDA exposed outside (Additional file 1: Figure S2A).

Glycol chitosan (GCS) has been widely applied in biomedical field because of its outstanding water solubility as well as the pH-sensitive charge reversal property [36–38]. To ameliorate the in vivo behavior of PDA@GNRs, the PDA@GNRs was further functionalized with the GCS. This combination was due to the fact that the amine groups in GCS could react with the quinone groups in PDA via the Schiff-base reaction with the addition of EDC and NHS, so the free amino groups in GCS could easily exposed in the solution [39, 40]. To prove that GCS was successfully grafted on PDA@GNRs, the FTIR spectrum of PDA@GNRs and GCS-PDA@GNRs was compared (Additional file 1: Figure S1B). From the spectrum of GCS-PDA@GNRs, it could be seen that the peak intensity of C=O (1730 cm^−1^) for PDA decreased acutely after the Schiff base reaction, but no C=N was identifiable probably due to the fact that its absorbance was obscured by the C–H frame vibration. Meanwhile, a new slight peak at 1155 cm^−1^ (consistent with the C–O–C for GCS) was observed in the spectrum of GCS-PDA@GNRs, and a similar spectral peak at 1146 cm^−1^ was observed for the GCS sample. All of these gave a powerful evidence for the successful synthesis of GCS-PDA@GNRs. Compared with the TEM image of PDA@GNRs, the average length and diameter had no obvious change after being modified with GCS [Fig. 1a (a3)]. Under the same concentration of GNRs, the longitudinal plasmon band maxima had an obvious rise and no significant red shift occurred after being modified with GCS (Fig. 1b). Presumably, the increase of longitudinal plasmon band maxima could be contributed to the hydrogen-bond interactions between PDA and GCS, facilitating the motion of electrons [41, 42]. It is reported that the free amine groups on the GCS backbones exposing in the acidic solution could be protonated, leading to a reversal of surface charge from negative value to positive value [36, 38]. Additional file 1: Figure S2B plotted a predictable phenomenon that the zeta potential of GCS-PDA@GNRs increased gradually from a negative charge (-6.7 mV) at pH 8.0 to a positive charge (13.7 mV) at pH 6.0. Therefore, variations on surface charge value of GCS-PDA@GNRs could be used as a parameter to evidence whether the PDA@GNRs had been successfully modified with GCS.

The catechol groups in PDA have been confirmed to be able to chelate metal ions [43] and reveal a pH-responsive release ability [44]. As we all know, silver ions (Ag^+^ ions) has a wide spectrum antimicrobial performance via different killing mechanisms (e.g., membrane damage and disturbance of cell metabolism triggered though the high reactive oxygen species production) [5, 45], and such properties made Ag^+^ ions be loaded onto PDA@GNRs as a promising drug in the treatment of abscess. Because of the reductive ability of PDA [46], we modified GCS on PDA@GNRs earlier to suppress Ag^+^ ions being reduced to silver nanoparticles (AgNPs) by PDA. Following GCS modification, Ag^+^ ions was loaded onto PDA@GNRs through the coordination of Ag^+^ ions with the oxygen atoms of the hydroxyl groups of catechol in PDA, forming the catechol-Ag complexes [43, 44]. Following synthesis and dialysis, the final product of Ag^+^-GCS-PDA@GNRs was analyzed by FTIR and UV–vis-NIR spectroscopy. Because the Ag^+^ ions had no infrared and ultraviolet absorption capacity, there was no obvious change in FTIR (Additional file 1: Figure S1B) as well as UV–vis-NIR spectrum (Fig. 1b) after being loaded with Ag^+^ ions. No surface plasmon resonance (SPR) peak of silver NPs at around 400 nm was observed from the UV–Vis-NIR spectra of Ag^+^-GCS-PDA@GNRs [47], which further proved that the Ag^+^ ions was not reduced to AgNPs by PDA. In this study, ICP-MS was used to determine the capacity of GCS-PDA@GNRs for Ag^+^ ions loading and confirm the successful synthesis of Ag^+^-GCS-PDA@GNRs. As shown in Fig. 1C, the loading amount of Ag^+^ ions raised with the concentration of silver ions increasing. Upon the concentrations of silver ions increased to 100 µg/mL, 9.9 µg silver ions was loaded on GCS-PDA@GNRs. Only a slight raise in Ag^+^ ions loading was observed by further increasing the concentration of Ag^+^ ions to 125 or 150 µg/mL. Therefore, in this study, we chose the 100 µg/mL of Ag^+^ ions as the concentration for silver loading on the GCS-PDA@GNRs. In this situation, Ag^+^-GCS-PDA@GNRs gave a mass ratio of Ag^+^ ions and GNRs as ~ 1: 23. After careful calculation, the embedding ratio of Ag^+^ ions was ~ 77.7% and the molar ratio close to 50:1 of Ag^+^ ions and GNRs within the Ag^+^-GCS-PDA@GNRs. As we expected, the PDA@GNRs had a high loading efficiency of Ag^+^ ions through our preparation method. Figure 1a (a4) showed the high-magnification TEM images of Ag^+^-GCS-PDA@GNRs, which exhibited that no significant variation of the average length and diameter occurred after being loaded with silver ions as well as no AgNPs was observed in the high-magnification TEM image of Ag^+^-GCS-PDA@GNRs.

After being modified with GCS and Ag^+^ ions, the Ag^+^-GCS-PDA@GNRs should possess both the pH-responsive charge reversal ability and the pH-sensitive release capacity. In order to verify whether the Ag^+^-GCS-PDA@GNRs had the similar pH-responsive charge switching property as GCS-PDA@GNRs, the zeta potential of Ag^+^-GCS-PDA@GNRs at different PH values (pH, 8.0–6.0) was measured (Fig. 1d). It was observed that a gradual change of the zeta potential of Ag^+^-GCS-PDA@GNRs from a negative charge (− 4.9 mV) at pH 8.0 to a net-positive one (14.7 mV) as pH values were decreased, which plotted a predictable result that the inversion of its surface charge depended on their local pH environment. To evaluate the pH-dependent release behavior of the loaded Ag^+^ ions from the PDA coated GNRs, the Ag^+^-GCS-PDA@GNRs were diluted in PB each under different pH values (pH = 7.4 and 6.3) at 37 °C in darkness for predetermined time points, and with laser irradiation at pH 6.3 for the specific time points (0, 10 min, 1 and 4 h). The release profiles of Ag^+^ ions were determined by ICP-MS (Fig. 1e). Only ~ 10% of the loaded Ag^+^ ions was released at pH 7.4 within 8 h. Nevertheless, the loaded Ag^+^ ions from Ag^+^-GCS-PDA@GNRs exhibited a favorable high-efficiency release behavior from the PDA@GNRs in PB at pH 6.3, which demonstrated that ~ 21.2% of the loaded Ag^+^ ions was released in the initial 1 h with a high release rate. The release value of Ag^+^ ions then neared 38.2% of the total loaded Ag^+^ ions upon 4 h immersion, and kept growing slowly until 44.1% of the loaded Ag^+^ ions in 8 h. The pH-sensitive release behavior of Ag^+^ ions was probably due to the hydrogen ions (H^+^) in PB buffer (pH = 6.3) could interact with the lone electron pairs of the oxygen ions in the hydroxyl groups of catechol, leading to the detachment of Ag^+^ ions from the PDA@GNRs [29, 48]. It’s worth noting that Ag^+^-GCS-PDA@GNRs exhibited an accelerated drug release behavior, reaching ~ 80% cumulative loaded Ag^+^ ions release over 8 h with laser irradiation. Since the Ag^+^-GCS-PDA@GNRs could efficiently convert NIR laser into thermal energy, the laser irradiation significantly facilitated the release of Ag^+^ ions from Ag^+^-GCS-PDA@GNRs in PB buffer (pH = 6.3) by increasing the temperature. The above results confirmed our previous speculation that our bactericidal depot was able to achieve the Ag^+^ ions controllable release at specific bacterium-targeted sites with minimal premature leakage, which could avoid the release of Ag^+^ ions in normal tissue.

### Photothermal conversion properties of Ag^+^-GCS-PDA@GNRs

Near-infrared (NIR) region (650–900 nm) is widely used to photothermal therapy (PTT) owing to their specific tumor therapeutic efficacy and reduced side effects to normal tissues [49]. GNRs have been considered to be one of the most efficient drug delivery carriers for NIR photothermal therapy due to its large surface area and ability to absorb NIR light irradiation and to release it as heat [50, 51]. Therefore, combined with the high photothermal conversion efficiency of GNRs, the targeted nanomaterial (GCS-PDA@GNRs) loaded with Ag^+^ ions might be able to further enhance its therapy effect of abscess. Since the longitudinal LSPR peak of Ag^+^-GCS-PDA@GNRs was 810 nm, thus an 808 nm laser was selected to appraise the photothermal conversion abilities of Ag^+^-GCS-PDA@GNRs in PB buffer (pH 6.3) under the preceding experimental parameters in experimental section. As shown in Fig. 1f, the heating profiles observed for Ag^+^-GCS-PDA@GNRs suspension irradiated at 0.5 W cm^−2^, and a similar heating profile at 0.25 W cm^−2^ was observed in Additional file 1: Figure S4, which illustrated that the photothermal conversion efficiency of Ag^+^-GCS-PDA@GNRs was depended on its concentration and laser power irradiation. The higher concentration of Ag^+^-GCS-PDA@GNRs, the higher temperature of its suspensions was observed. Additionally, all the concentrations of Ag^+^-GCS-PDA@GNRs suspensions showed a consistent regular that which had a higher temperature at 0.5 than 0.25 W cm^−2^. The temperature of Ag^+^-GCS-PDA@GNRs suspension (11.7–46.6 μg/mL) reached plateaus within 7 min with the irradiation at 0.5 W cm^−2^, and the corresponding thermographic images were shown in Fig. 1g. While, the emergence of temperature plateaus of Ag^+^-GCS-PDA@GNRs suspension (11.7–46.6 μg/mL) required a longer period of irradiation of 9 min at 0.25 W cm^−2^. An obvious temperature increment of Ag^+^-GCS-PDA@GNRs suspensions (46.6 μg/mL) was observed at the first 3 min until a saturation temperature about 48.87 and 61.57 °C with the irradiation at 0.25 and 0.5 W cm^−2^ for 11 min, respectively. However, the temperature increment of PB buffer was negligible under the same condition (Additional file 1: Figure S4; Fig. 1f). These results proved that Ag^+^-GCS-PDA@GNRs had excellent photothermal conversion efficiency, which renders the nanomaterial very promising as an efficient and benign chemo-photothermal combined antimicrobial for abscess. Meanwhile, the Ag^+^-GCS-PDA@GNRs showed excellent photostability, with less than 6% reduction in absorption and constant photothermal conversion efficiency after five cycles of laser irradiation at 0.5 W cm^−2^ (Fig. 1h, i). Heat at temperatures above 50 °C causes the irreversible eradiation of bacteria by destruction of their proteins/enzymes and inhibition of their essential intracellular reactions [13, 52]. Considering the destructive effect of Ag^+^ ions and temperatures to bacteria, the 23.3 μg/mL of concentration of Ag^+^-GCS-PDA@GNRs combined with the power intensity of 0.5 W cm^−2^ for 7 min was selected to study the following chemo-photothermal combinational antibacterial effect.

### pH-dependent bacterium-specific interactions of Ag^+^-GCS-PDA@GNRs

**Fig. 2:**
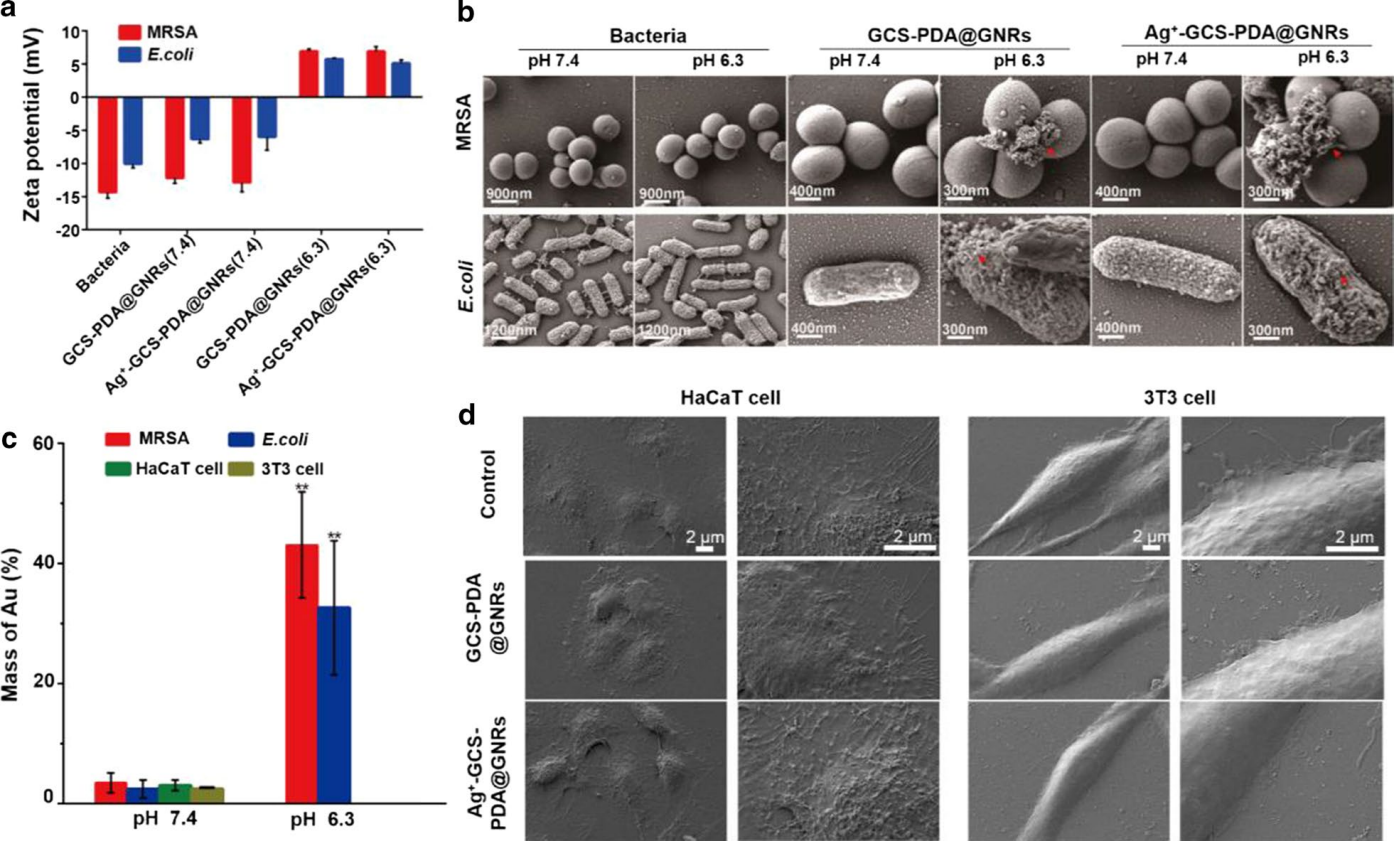
**a** Zeta potential of bacteria before and after incubation with GCS-PDA@GNRs and Ag^+^-GCS-PDA@GNRs at pH 6.3 or pH 7.4. SEM images of (**b**) bacteria (pH 6.3 & pH 7.4) and (**d**) HaCat cells and NIH 3T3 fibroblasts (pH 7.4) before and after incubation with GCS-PDA@GNRs and Ag^+^-GCS-PDA@GNRs. The red arrows indicated the GNRs. **c**The amount of NP bound on the bacteria at different pH conditions (pH 7.4 and 6.3) after 4 h co-incubation with Ag^+^-GCS-PDA@GNRs and on the cells (pH 7.4) after 24 h co-incubation with Ag^+^-GCS-PDA@GNRs. The significant difference was compared between the bacteria at pH 7.4 condition and at pH 6.3 condition. * and ** present *p *< 0.05 and *p *< 0.01, respectively

Generally, bacterial cells are negatively charged attributed to the high proportion of anionic phospholipids on their cell walls [53]. As indicated in Fig. 1d and Additional file 1: Figure S2B, Ag^+^-GCS-PDA@GNRs and GCS-PDA@GNRs have unique surface charge switching that depends on their local pH, which exhibits a slight negative charge at pH 7.4 while presenting a positive charge at pH 6.3. Thus, we speculate that GCS-PDA@GNRs and Ag^+^-GCS-PDA@GNRs can participate in bacterium-specific interactions in the acidic pus of focal infections (pH 6.3) while minimizing their direct contact with neighboring host cells (pH 7.4). To confirm our speculation, the as-prepared nanomaterials were incubated with MRSA and *E. coli* bacteria for 30 min at 37 °C, respectively. Upon contacting with GCS-PDA@GNRs or Ag^+^-GCS-PDA@GNRs at pH 6.3, bacterial cells were rapidly sedimented to the bottom of tubes within 15 min, which was possibly attributed to strong electrostatic attraction between positively-charged nanomaterial and negatively-charged bacterial membranes. However, such phenomenon was not observed under pH 7.4 condition, which seems to show that our bactericides hybrid possess a pH-dependent specificity for bacterial cells. The following zeta potential results and SEM analysis provided more supportive evidence on the acidity-triggered bacterium-targeting properties of GCS-PDA@GNRs or Ag^+^-GCS-PDA@GNRs. As presented in Fig. 2a, zeta potential measurements indicated that MRSA and *E. coli* had a negative surface charge. In the acidic environment (pH 6.3), the GCS-PDA@GNRs and Ag^+^-GCS-PDA@GNRs showed a net-positive surface charge that resulted in strong electrostatic interactions with the negatively-charged bacterial membranes of MRSA and *E. coli*, converting the bacterial surface charge from negative to positive. However, the negative charge of cell membranes of MRSA and *E. coli* was still observed after co-incubated with GCS-PDA@GNRs or Ag^+^-GCS-PDA@GNRs in the physiological environment (pH 7.4). Moreover, the SEM images showed that a large number of nanomaterials (GCS-PDA@GNRs or Ag^+^-GCS-PDA@GNRs) were closely bound on the surfaces of both MRSA and *E. coli* bacteria cells in the acidic environment (pH 6.3), while such phenomenon was not observed in the physiological environment (pH 7.4) (Fig. 2b). After co-incubation with nanomaterials with bacteria, the quantitative analysis determined from ICP-MS showed that 43.1 and 32.6% of nanomaterials adhered on the MRSA and *E. coli* bacteria cells at pH 6.3 condition, respectively; while the amount was less than 3.5% at pH 7.4 condition (Fig. 2c).

On the other hand, when exposed to 3T3 fibroblasts at pH 7.4 (the pH of physiological environment), the test GCS-PDA@GNRs and Ag^+^-GCS-PDA@GNRs had a slight negative charge (Fig. 1d and Additional file 1: Figure S2B), resulting in no or slightly adhesion to the negatively-charged host cells (Fig. 2d). Consistently, the quantities of nanomaterial bound on cells were only 3.1 and 2.5% of total adding amount after 24 h for HaCaT cells and 3T3 fibroblasts, respectively (Fig. 2c). These above results reveal that the GCS-PDA@GNRs and Ag^+^-GCS-PDA@GNRs can be used to achieve various bacteria-specific aggregations in the acidic setting of a focal infection.

### In vitro antimicrobial activity

**Fig. 3:**
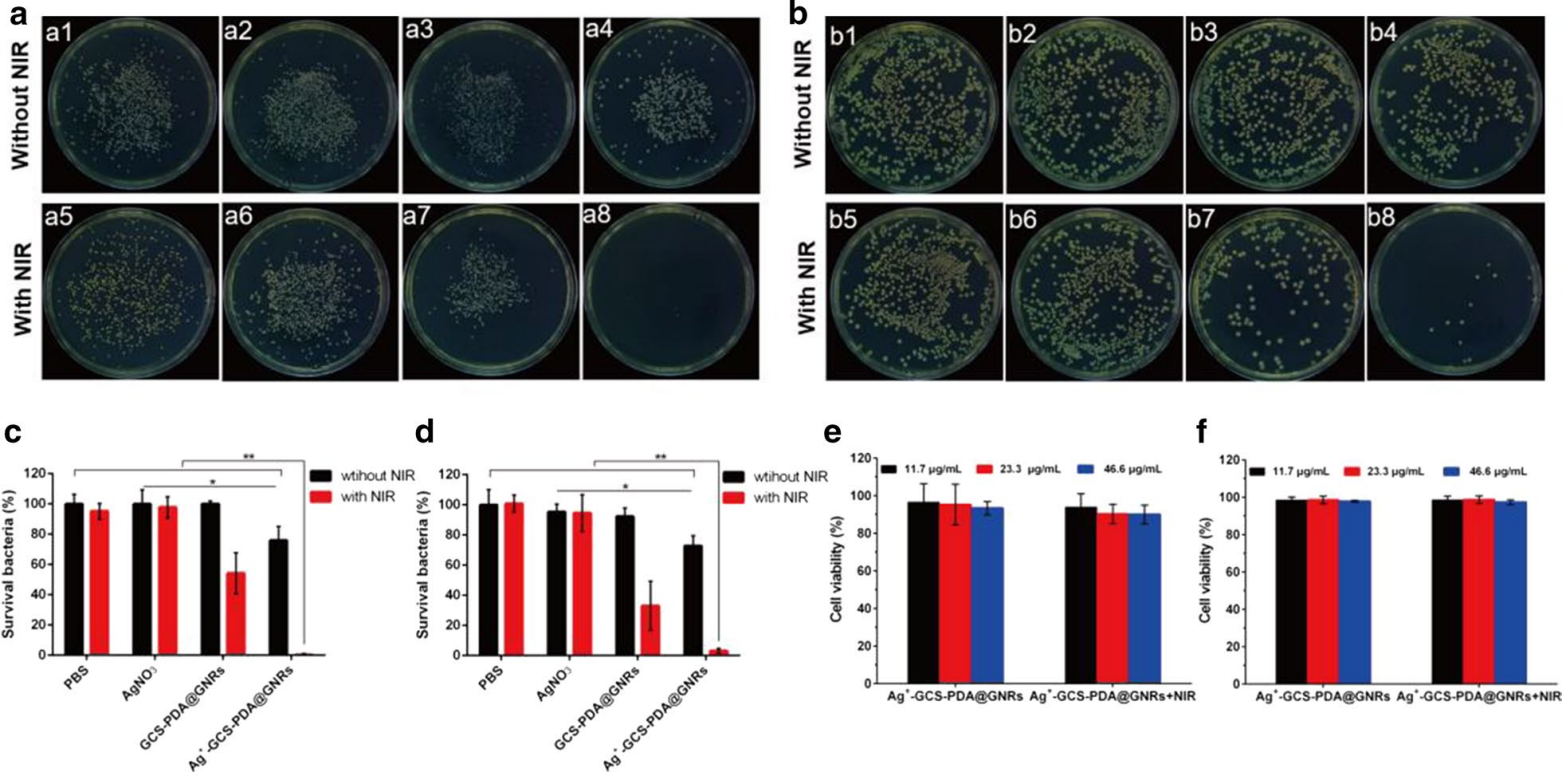
*In vitro* chemo-photothermal combinational therapy for bacteria eradicating. Representative images of bacterial colonies formed by (**a**) MRSA and (**b**) *E. coli* after exposed to (a1, b1) PBS, (a2, b2) AgNO_3_, (a3, b3) GCS-PDA@GNRs, (a4, b4) Ag^+^-GCS-PDA@GNRs, (a5, b5) PBS + NIR, (a6, b6) AgNO_3_ + NIR, (a7, b7) GCS-PDA@GNRs + NIR and (a8, b8) Ag^+^-GCS-PDA@GNRs + NIR, respectively. The bacterial viability of (**c**) MRSA and (**d**) *E. coli* after treatment of PBS, AgNO_3_, GCS-PDA@GNRs and Ag^+^-GCS-PDA@GNRs with or without NIR laser, respectively. The cell viability of (**e**) HaCaT cells and (**f**) 3T3 fibroblasts treated with different concentrations of Ag^+^-GCS-PDA@GNRs with or without NIR light irradiation (0.5 W cm^−2^, 7 min). * and ** present *p *< 0.05 and *p *< 0.01, respectively

**Fig. 4:**
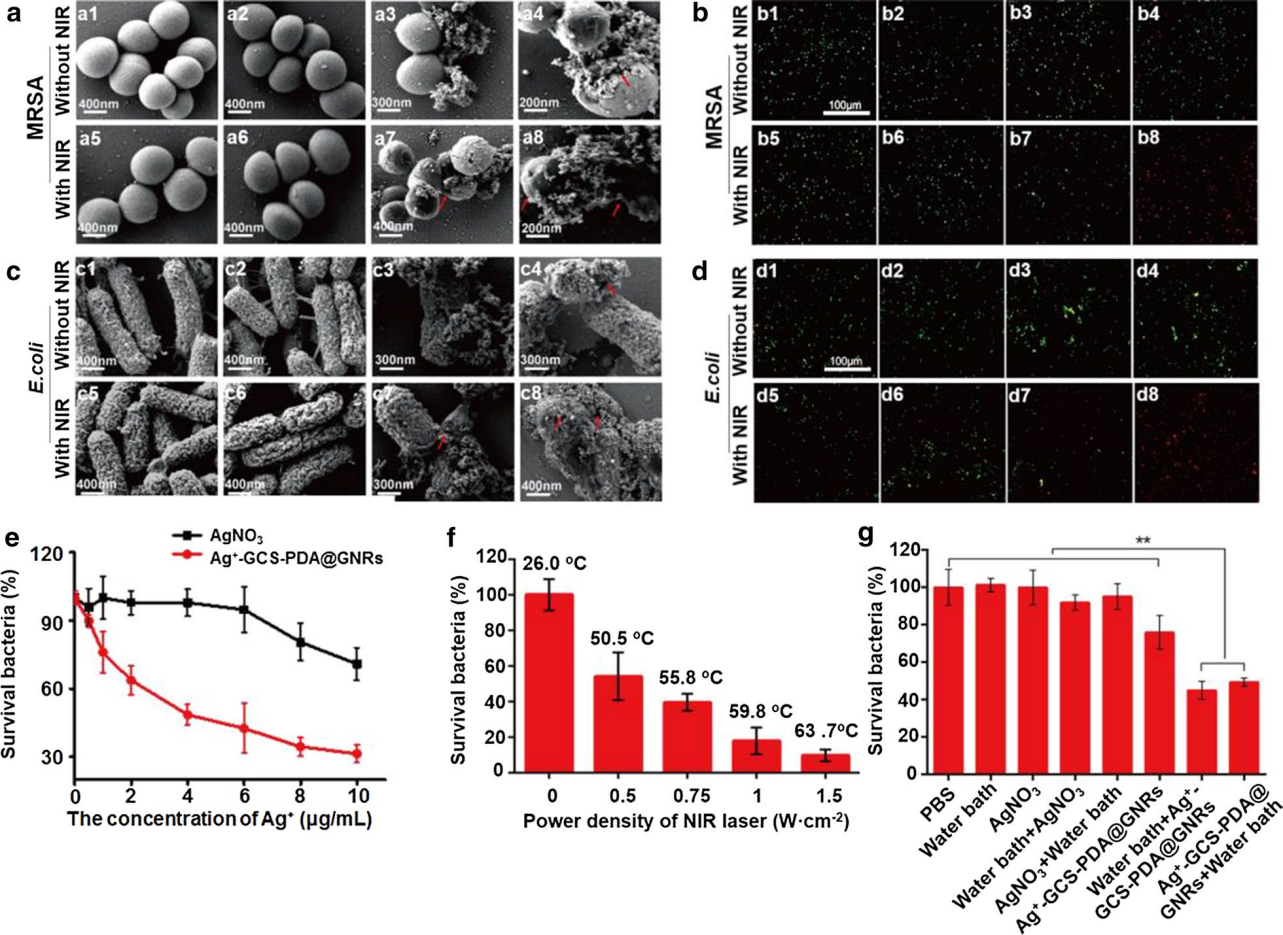
SEM images of (**a**) MRSA (pH 6.3) and (**c**) *E. coli* (pH 6.3) treated with (a1, c1) PBS, (a2, c2) AgNO_3_, (a3, c3) GCS-PDA@GNRs, (a4, c4) Ag^+^-GCS-PDA@GNRs, (a5, c5) PBS + NIR, (a6, c6) AgNO_3_ +NIR, (a7, c7) GCS-PDA@GNRs + NIR and (a8, c8) Ag^+^-GCS-PDA@GNRs + NIR, respectively. The red arrows indicate the lesions and collapse of bacteria. Images of Live/Dead staining assay for (**b**) MRSA (pH 6.3) and (**d**) *E. coli* (pH 6.3) treated by (b1, d1) PBS, (b2, d2) AgNO_3_, (b3, d3) GCS-PDA@GNRs, (b4, d4) Ag^+^-GCS-PDA@GNRs, (b5, d5) PBS + NIR, (b6, d6) AgNO_3_ + NIR, (b7, d7) GCS-PDA@GNRs + NIR and (b8, d8) Ag^+^-GCS-PDA@GNRs + NIR, respectively. **e** Bacterial viability of MRSA treated by different dose of AgNO_3_ and Ag^+^-GCS-PDA@GNRs for 4 h. **f** Bacterial viability of MRSA treated with Ag^+^-GCS-PDA@GNRs under NIR laser (0.5 W cm^−2^, 7 min) of different power density. **g** Bacterial viability of MRSA treated by water bath (50 °C) before and after combinational therapy of AgNO_3_ or Ag^+^-GCS-PDA@GNRs. * and ** present *p *< 0.05 and *p *< 0.01, respectively

To investigate the in vitro chemotherapeutic effect of our bactericides hybrid, the drug-resistance Gram-positive MRSA or Gram-negative *E. coli* were co-cultured with Ag^+^-GCS-PDA@GNRs at a concentration of 23.3 µg·mL^−1^ in PBS (pH 6.3) for 4 h, using the pristine untreated, GCS-PDA@GNRs and AgNO_3_ as controls. As shown in Fig. 3a–d, the percentage of bacteria survival in AgNO_3_ and GCS-PDA@GNRs treated groups was all above 92%; while for the Ag^+^-GCS-PDA@GNRs treated groups, only 76.0 and 72.8% bacteria were survived for MRSA and *E. coli*, respectively. These results indicated that Ag^+^-GCS-PDA@GNRs showed higher antibacterial efficiency than GCS-PDA@GNRs or AgNO_3_ alone with an equivalent amount of Ag^+^ ions. The low antibacterial efficiency of AgNO_3_ used here was probably because the toxicity of Ag^+^ ions from AgNO_3_ was considerably suppressed by the dissolved chloride ions in PBS, whereas the in situ released Ag^+^ ions from Ag^+^-GCS-PDA@GNRs triggered by the acid circumstance could directly interact with the cell walls of bacteria to combat with bacteria, resulting in improvement the local concentration of Ag^+^ ions [5, 7, 10]. The dose-variant antibacterial behavior of Ag^+^ ions against MRSA was further confirmed the high antibacterial activity of Ag^+^-GCS-PDA@GNRs (Fig. 4e). Specially, a quite high concentration of Ag^+^ ions (10 µg/mL) from AgNO_3_ was required to reach the similar antibacterial effect of Ag^+^-GCS-PDA@GNRs (Ag^+^: 1 µg/mL). These results suggested that the Ag^+^-GCS-PDA@GNRs could largely reduce the dose of Ag^+^ ions via maximizing the utilization of Ag^+^ ions to enhance the bactericidal efficacy, which might alleviate the risks of excess silver for application in the treatment of bacterial infection [7]. However, even a high concentration of the Ag^+^-GCS-PDA@GNRs still could not totally kill MRSA bacteria (survival bacteria: 31.4%).

To further improve the antibacterial effect, PTT was introduced to construct the chemo-photothermal combinational bactericides system. Thereby, the test samples were further irradiated with an 808 nm-laser at 0.5 W cm^−2^ for 7 min. For the Ag^+^-GCS-PDA@GNRs + NIR treated group, the bacterial inactivation percentages were 99.6 and 96.8% for MRSA and *E. coli*, respectively (Fig. 3a–d). To further verify the enhanced antibacterial effect of the chemo-photothermal combinational therapy, the bactericidal activity of GCS-PDA@GNRs-based PTT was studied. Thus, the MRSA bacterial viability in GCS-PDA@GNRs group with different power density of NIR irradiation was further investigated. As presented in Fig. 4f, the survival rate of bacteria was still up to 9% even when enhancing the power density of NIR to 1.5 W cm^−2^ (7 min, 63.7 °C). Therefore, compared with the monotherapy of chemo or photothermal, chemo-photothermal synergistic therapy of Ag^+^-GCS-PDA@GNRs showed remarkable bactericidal activity, which not only could reduce the dose of silver but also decrease the temperature used in PTT.

To deeply clarify the antibacterial behavior, SEM observation and Live/Dead staining assay were used. As shown in Fig. 4a, c, the morphology of bacteria treated with GCS-PDA@GNRs or AgNO_3_ alone was similar to that with untreated group, which were typically spherical-shaped (for MRSA) or rod-shaped (for *E. coli*) with smooth and intact cell membranes. However, after being treated with Ag^+^-GCS-PDA@GNRs, lesions and collapse were observed on the surface of MRSA or *E. coli*. The results obtained from Live/Dead staining assay also certified these. Live bacteria with intact cell membranes will be stained green, while dead/damaged bacteria with destructive membranes will be stained red. As presented in Fig. 4b, d, the AgNO_3_ or GCS-PDA@GNRs alone treated bacteria showed totally green fluorescence, while a lot of red signal was observed for Ag^+^-GCS-PDA@GNRs treated bacteria. These results were probably due to the Ag^+^-GCS-PDA@GNRs directly binding with the cell walls of bacteria in the acid environment could in situ release Ag^+^ ions, leading to cell wall and membrane disruption. After combined with PTT, the bacteria treated with PBS + NIR or AgNO_3_ + NIR still showed clear edges and smooth bodies, while the wrinkled and collapsed surfaces were found on the bacteria treated with GCS-PDA@GNRs + NIR, indicating that the hyperthermia (> 50 °C) could damage the bacterial cell (Fig. 4a, c) [5]. However, seriously collapsed and split membranes were clearly seen in large quantities of bacteria in Ag^+^-GCS-PDA@GNRs + NIR treated group, indicating highly enhanced bactericidal effect of Ag^+^-GCS-PDA@GNRs via chemo-photothermal synergistic therapy. The Live/Dead staining data also supported these (Fig. 4b, d). Consequently, the above results confirmed that the Ag^+^-GCS-PDA@GNRs was a superior chemo-photothermal synergistic system with outstanding antibacterial efficacy in the presence of low-concentrated Ag^+^ ions.

Furthermore, the probable mechanism of the chemo-photothermal combinational therapy based on Ag^+^-GCS-PDA@GNRs was discussed. One of the possible mechanism is that Ag^+^-GCS-PDA@GNRs could specifically target and in situ release the Ag^+^ ions to the cell membranes of bacteria due to its acidity-triggered Ag^+^ ions release and charge reversal properties, which could disrupt the bacterial membranes and improve the permeability and sensitivity to heat [54, 55]. On the other hand, the hyperthermia caused by PTT could promote the Ag^+^ ions release (Fig. 1e) that may further damage bacteria cells. To demonstrate our hypothesis, model bacteria MRSA were treated and divided by the following groups: (1) Bacteria were cultured in PBS (pH, 6.3) at 50 °C using a water bath for 10 min (water bath group); (2) Bacteria were treated with AgNO_3_ alone (AgNO_3_ group); (3) Bacteria were cultured in PBS (pH, 6.3) at 50 °C using a water bath for 10 min firstly, followed by another treatment of AgNO_3_ (water bath + AgNO_3_ group) for 4 h; (4) Bacteria were firstly treated with AgNO_3_ for 4 h, and then incubated at 50 °C bath for 10 min (AgNO_3_ + water bath). (5) Bacteria were treated with Ag^+^-GCS-PDA@GNRs alone (Ag^+^-GCS-PDA@GNRs group); (6) Bacteria were cultured in PBS (pH, 6.3) at 50 °C using a water bath for 10 min firstly, followed by another treatment of Ag^+^-GCS-PDA@GNRs (water bath + Ag^+^-GCS-PDA@GNRs group) for 4 h; (7) Bacteria were firstly treated with Ag^+^-GCS-PDA@GNRs for 4 h, and then incubated at 50 °C bath for 10 min (Ag^+^-GCS-PDA@GNRs + water bath). The bacteria without any treatment were regarded as control group. As shown in Fig. 4g, no significant difference of bacterial viability was observed between the control and the 50 °C water bath group (*p *> 0.05), suggesting that external heating had no bactericidal activity [15]. Nevertheless, compared with the Ag^+^-GCS-PDA@GNRs group, the water bath + Ag^+^-GCS-PDA@GNRs group and Ag^+^-GCS-PDA@GNRs + water bath group presented an significantly enhanced bactericidal efficiency (*p *< 0.05), indicating that the pretreatment of heat could promote Ag^+^ ions release to kill bacteria, while pretreatment of Ag^+^-GCS-PDA@GNRs was helpful in strengthening the bactericidal efficacy of the following thermal therapy. Based on the above data, we speculated that Ag^+^-GCS-PDA@GNRs destroyed bacteria via synergistic mechanisms as follows. Firstly, the Ag^+^-GCS-PDA@GNRs could directly target to the surface of bacterial cell by the electrostatic attraction (Fig. 2b) and in situ release Ag^+^ ions to disrupt the cell membrane [7] (Fig. 4a–d). Next, as the ruptured membrane have improved sensitivity and permeability to heat [55], combined PTT could ablate bacteria by denaturizing the proteins/enzymes of bacteria when the temperature reached to 50 °C [13]. Furthermore, the heat derived from PTT was able to promote release of Ag^+^ ions (Fig. 1e), which would lead to more Ag^+^ ions enter into the bacterial cell body through the damaged membrane and further cause DNA damage and perturbation of cell metabolism [9, 56]. Overall, all these results confirmed our bactericidal depot possessed outstanding bactericidal activity through the chemo-photothermal synergistic effect.

For the assessment of in vitro cytotoxicity of Ag^+^-GCS-PDA@GNRs, the HaCaT cells and 3T3 fibroblasts were co-cultured with a range of concentration of Ag^+^-GCS-PDA@GNRs (11.7, 23.3 and 46.6 µg/mL) for 24 h, and the viability of cells was evaluated using a cell counting kit-8 (CCK-8) assay. As presented in Fig. 3e, f, the cell viability of HaCaT cells and 3T3 fibroblasts co-cultured with different concentrations of Ag^+^-GCS-PDA@GNRs whether exposed to NIR light (0.5 W cm^−2^, 7 min) or not was high and showed no significant difference, indicating that Ag^+^-GCS-PDA@GNRs alone and even combined with NIR light irradiation exhibited no toxicity to healthy tissues. This was possibly because the test pH-responsive Ag^+^-GCS-PDA@GNRs showed a net neutral charge and seldom Ag^+^ ions release at pH 7.4 (Fig. 1d, e), so the adherence of nanomaterial to the negatively-charged host cells and the toxicity of Ag^+^ ions could be effectively avoided, which was a similar observation as the previous works [19]. In addition, the hemolytic property of Ag^+^-GCS-PDA@GNRs on human erythrocytes was also investigated. As shown in Additional file 1: Figure S5, Ag^+^-GCS-PDA@GNRs did not damage erythrocytes even if the dose of the nanomaterial increased up to the twofold of treatment dose (hemolysis ratio % < 5%) [57]. This result demonstrated the biosafety of Ag^+^-GCS-PDA@GNRs in blood circulation.

Collectively, all the results demonstrated that the chemo-photothermal synergistic therapy of Ag^+^-GCS-PDA@GNRs had competitive advantage over monotherapy of silver or PTT in vitro. This system exhibited much enhanced broad-spectrum antibacterial activity compared to equivalent amount of AgNO_3_ solution or equivalent power density of PTT, which could not only reduce the side effects in vitro and in vivo due to the infusing minimum Ag^+^ ions, but also avoid the potential skin damage caused by the high power density of NIR laser.

### In vivo biodistribution of Ag^+^-GCS-PDA@GNRs

Firstly, in order to study the in vivo distribution of our antibacterial depot, Ag^+^-GCS-PDA@GNRs NPs were labeled with CY5-SE fluorescence agents (*f*-Ag^+^-GCS-PDA@GNRs). CY5-SE was speculated to bind with amine groups on GCS to form an irreversible covalent bond [58]. Compared with the absorption peak of Ag^+^-GCS-PDA@GNRs, an obvious absorption peak and fluorescent emission peak of *f*-Ag^+^-GCS-PDA@GNRs was detected at 650 nm and 670 nm (excited at 649 nm), which corresponded to the absorption and emission peak of CY5-SE (Additional file 1: Figure S3A, B), respectively. This gives a powerful evidence of successful conjugation of CY5-SE and GCS.

**Fig. 5:**
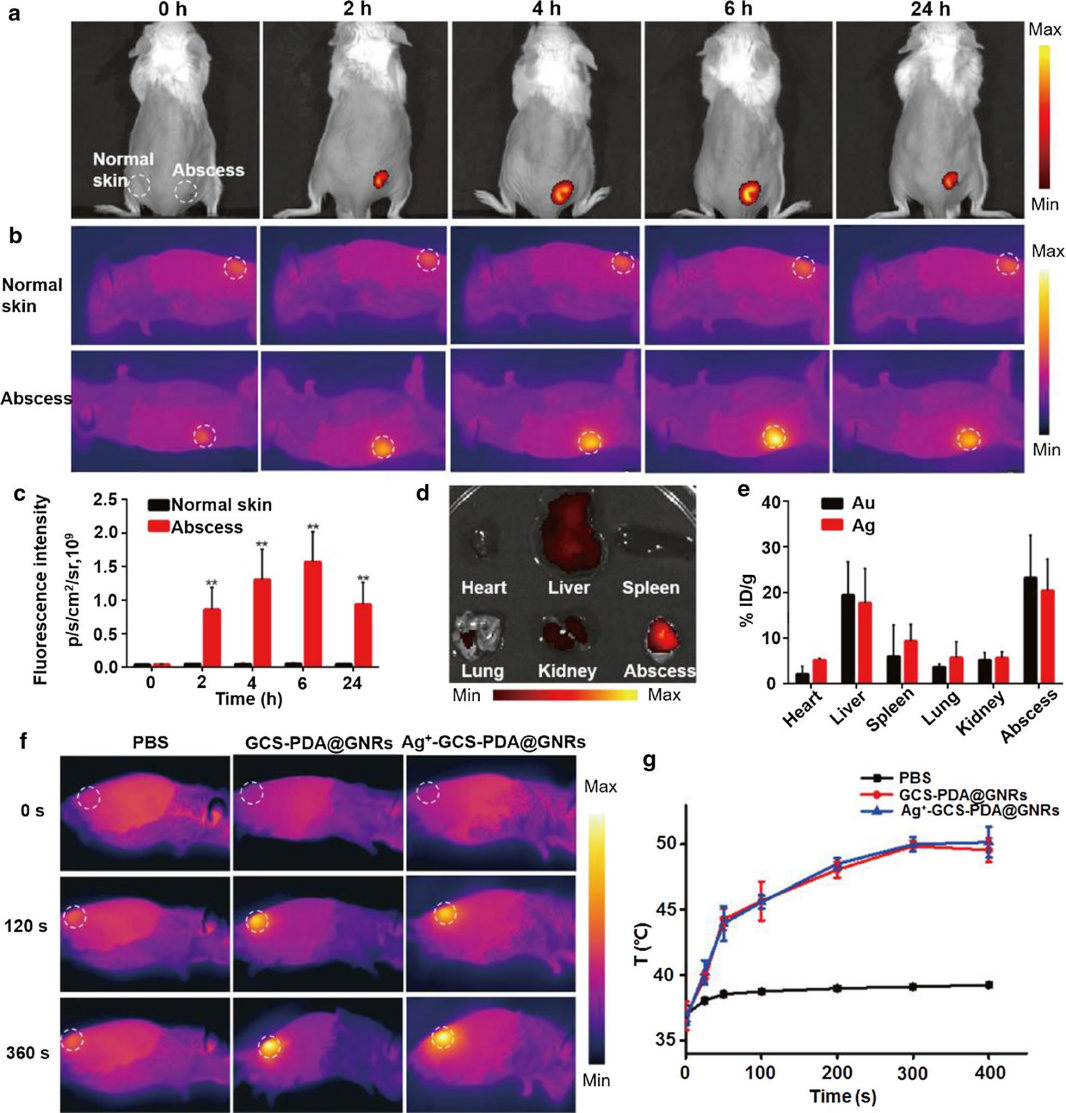
**a** *In vivo* NIRF images and (**b**) thermographic images of the mice bearing abscess intravenously injected with *f*-Ag^+^-GCS-PDA@GNRs at a dose of 4.7 mg/Kg at 0, 2, 4, 6 and 24 h post-injection. For the thermographic images, the test mice were irradiated under 808 nm near-infrared light (0.8 W cm^−2^, 6 min) at different time points after intravenous injection. **c** Corresponding NIRF intensities determined from Fig. 5a. **d** NIRF images of heart, liver, spleen, lung, kidney, and abscess extracted from the test mice intravenously injected with *f*-Ag^+^-GCS-PDA@GNRs at 24 h post-injection. **e** Quantitative amount of the Au and Ag in the major organs and abscess at 24 h post-injection. **f** Infrared thermography (0.8 W cm^−2^, 6 min) and (**g**) temperature elevations of the mice bearing abscess intravenously injected with Ag^+^-GCS-PDA@GNRs at a dose of 4.7 mg/Kg at 6 h post-injection. * and ** present *p *< 0.05 and *p *< 0.01, respectively

To evaluate whether the Ag^+^-GCS-PDA@GNRs could accumulate in the bacterial infection site, we monitored the mice bearing subcutaneous abscess at right flank treated with *f*-Ag^+^-GCS-PDA@GNRs using in vivo NIRF and thermographic imaging. As shown in Fig. 5a, c, after intravenously injected with *f*-Ag^+^-GCS-PDA@GNRs (4.7 mg/Kg), time-dependent fluorescence activation was only observed at the abscess site but was absent in the normal skin. The most obvious NIRF signal was detected at 6 h post-injection, and the signal still retained at abscess site after 24 h post-injection. In line with NIRF imaging results, the thermographic images also showed the potent hyperthermia at abscess site in a time-dependent manner (Fig. 5b). These results confirmed that *f*-Ag^+^-GCS-PDA@GNRs had a remarkable ability to achieve effective infected site accumulation, which probably due to the strong electrostatic interactions between the positively charged *f*-Ag^+^-GCS-PDA@GNRs and the negatively charged bacteria in the acidic focal infection area (pH 6.3). Moreover, the specific accumulation to infected site of designed *f*-Ag^+^-GCS-PDA@GNRs also suggested its potential capacity for in vivo imaging of bacterial infections as previously described [59].

Afterwards, the mice were sacrificed and the biodistributions of nanomaterial in major organs were detected using NIRF imaging analysis and ICP-MS. As shown in Fig. 5d, besides accumulated in abscess, our antibacterial depot was mainly presented in the liver, which was consistent with previous reports [60, 61]. Furthermore, quantitative data revealed that the percentage of injected dose per gram of tissue (% ID/g) in liver was 19.5% ID/g of Au and 17.7% ID/g of Ag, respectively; while the abscess accumulation of nanomaterial was 23.2% ID/g of Au and 20.4% ID/g of Ag, respectively (Fig. 5e). The percentage of Ag accumulated in the liver and abscess was slightly lower than that of Au, which was probably due to minimal Ag^+^ ions released in blood circulation (pH 7.4) as the in vitro released profile shown in Fig. 1e. These results further supported the NIRF imaging and thermographic imaging observations, indicating that the Ag^+^-GCS-PDA@GNRs indeed specifically targeted the infected site in vivo.

In order to evaluate the capacity of our antibacterial depot to produce in vivo hyperthermia at abscess, infected mice were intravenously injected with 100 µL of PBS, GCS-PDA@GNRs (4.7 mg/Kg) and Ag^+^-GCS-PDA@GNRs (4.7 mg/Kg), respectively, and followed by NIR irradiation under 808 nm (0–8 min, 0.8 W cm^−2^) at 6 h post-injection. The abscess temperature elevated to 49.9 and 50.0 °C for GCS-PDA@GNRs and Ag^+^-GCS-PDA@GNRs group, respectively, while no significant temperature increase was found in the PBS group (Fig. 5f, g). Distinctly, all these data implied that the Ag^+^-GCS-PDA@GNRs possessed remarkable chemo-photothermal synergistic effect in vivo.

### In vivo antibacterial activity of Ag^+^-GCS-PDA@GNRs

**Fig. 6:**
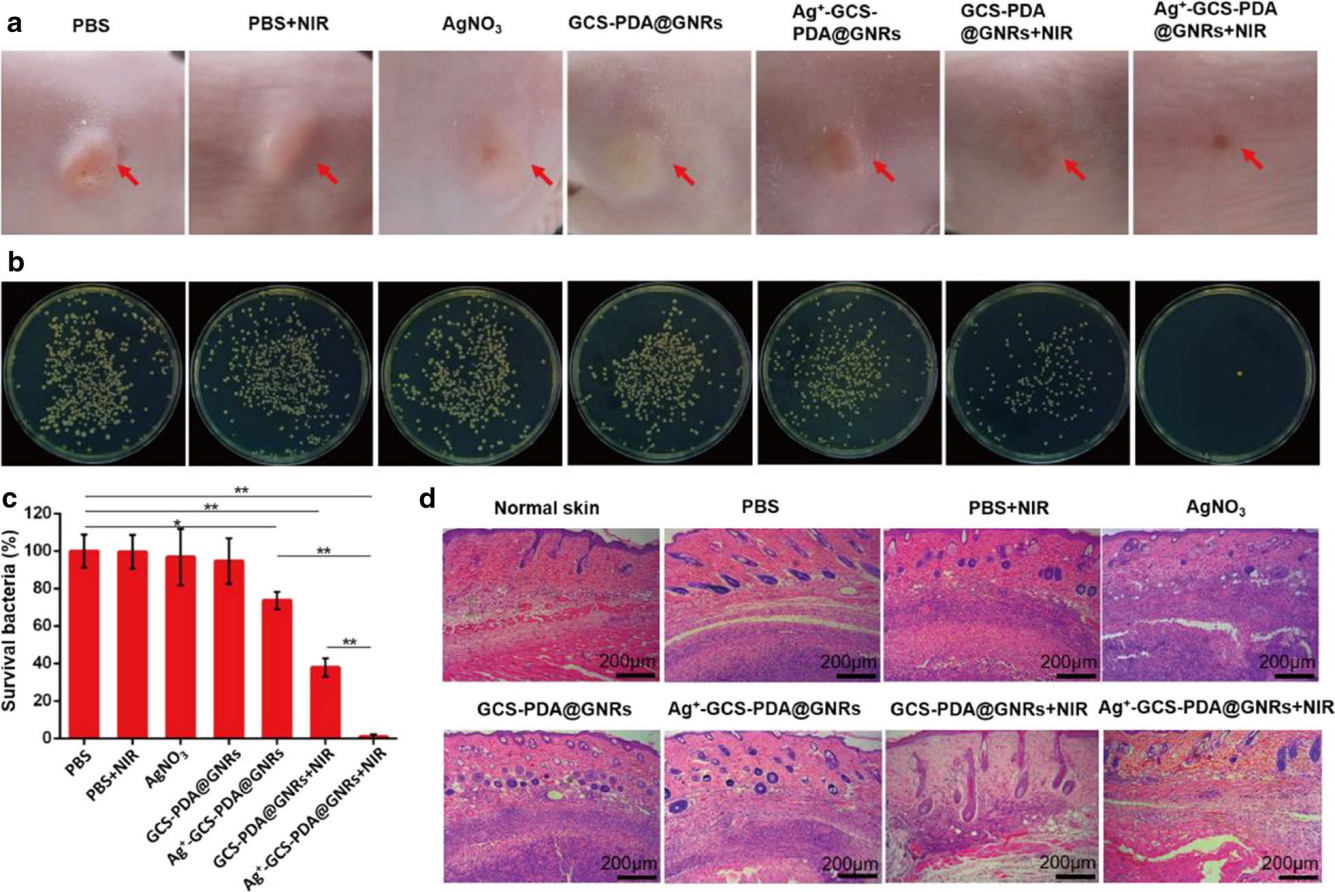
*In vivo* chemo-photothermal synergistic therapy for subcutaneous abscess. **a** Photographs of subcutaneous abscess on the dorsal surface of mice after 9 days treatment. **b** Photographs of bacterial CFUs and (**c**) corresponding quantitative results under various treatments. **d** Representative H&E staining images of infected skins that received various treatments. The red arrows indicate the subcutaneous abscess. * and ** present *p *< 0.05 and *p *< 0.01, respectively

The in vivo bactericidal effect of Ag^+^-GCS-PDA@GNRs was investigated using a murine subcutaneous abscess model. After the formation of subcutaneous abscess, the BALB/c mice were intravenously injected with 100 µL of PBS (10 mM, pH 6.3), AgNO_3_ (0.3 mg/Kg), GCS-PDA@GNRs (4.7 mg/Kg) and Ag^+^-GCS-PDA@GNRs (4.7 mg/Kg), respectively, and the mice in NIR groups were further exposed to 808 nm NIR irradiation (0.8 W cm^−2^, 6 min) at 6 h post-injection. As shown in Fig. 6a, after being treated for 9 days, the mice in Ag^+^-GCS-PDA@GNRs + NIR group showed no evident inflammation and abscess on the dorsal surface, while obvious abscess and red swelling of the skin were still observed on the tested mice in other groups. These results certificated that the strong bactericidal effects of chemo-photothermal therapy of Ag^+^-GCS-PDA@GNRs in vivo. In order to further quantify antibacterial efficacy of each test group in vivo, the infected tissues were homogenized and the bacteria colonies were counted using the standard plate counting assay. As indicated in Fig. 6b, c, the CFU counts in the groups that received PBS injection together with NIR or GCS-PDA@GNRs alone were similar to that in the PBS group (*p *> 0.05), while the CFU count in the Ag^+^-GCS-PDA@GNRs group was significantly less than that in the PBS group (*p *< 0.05), indicating that Ag^+^-GCS-PDA@GNRs exhibited antibacterial activity in vivo due to the in situ release of silver ions. In contrast, the GCS-PDA@GNRs + NIR group (SR: 37.9%) and Ag^+^-GCS-PDA@GNRs +NIR group (SR: 0.9%) showed a strong antibacterial effect, indicating the efficient antibacterial effect of PTT in treating abscess. More noteworthy is that the Ag^+^-GCS-PDA@GNRs +NIR group showed remarkable in vivo antibacterial activity (as their CFU count was reduced to only 0.9% of that of the control group, *p *< 0.05), and this reduction significantly exceeded that of the other groups (*p *< 0.05). This was probably because that the hyperthermia could not only kill bacteria, but also promote more Ag^+^ ions release from Ag^+^-GCS-PDA@GNRs to further fight against bacteria.

To further investigate the therapeutic effect, histological analysis in the model bacteria MRSA infected skin tissue was performed by hematoxylin and eosin (H&E) staining. As indicated in Fig. 6d, a huge abscess filled with necrotic tissues, infiltrated inflammatory cells and bacteria were clearly observed in the subcutaneous tissue obtained from samples of PBS, AgNO_3_, GCS-PDA@GNRs and PBS + NIR groups, revealing signs of sustained severe infection. Consistent with the CFU count data, the infectious area was reduced in the Ag^+^-GCS-PDA@GNRs group, which further indicated that Ag^+^-GCS-PDA@GNRs had antibacterial activity in vivo. However, obvious inflammation response in the subcutaneous tissue still existed in the Ag^+^-GCS-PDA@GNRs and GCS-PDA@GNRs +NIR groups, indicating that mono silver or PTT could not completely ablate the abscess. In contrast, a significant reduction of infiltration of inflammatory cells was observed following the treatment with Ag^+^-GCS-PDA@GNRs irradiated by NIR light, and the morphological features with blood vessels and hair follicles similar to the normal skin were also seen. This result further demonstrated the excellent synergistic therapeutic effect of Ag^+^-GCS-PDA@GNRs via chemo-photothermal therapy in treating subcutaneous abscesses. Notably, no obvious foreign-body reaction in the healthy tissues surrounded the abscess was observed in the Ag^+^-GCS-PDA@GNRs + NIR group on the 9th day post-treatment, suggesting no damage to surrounding healthy tissues. This further demonstrated that the pH-responsive Ag^+^-GCS-PDA@GNRs could specifically target to the acidic bacterial infected site (pH 6.3). Therefore, the chemo-photothermal synergistic therapy of Ag^+^-GCS-PDA@GNR is an effective and safe way to treat MRSA-infected subcutaneous abscess.

The overuse of classic antibiotics has caused emergence of multidrug-resistant bacteria, which leads to great threat to human health and gained heavy burden to public health care [62]. Thus, researchers have tried their efforts to develop alternative antibacterial agents such as silver materials, photothermal agents and photodynamic therapeutic agents to eliminate bacterial infections [13, 63]. For instance, Reithofer et al. [64] prepared a silver nanoparticles (AgNPs) impregnated hydrogel as a bactericidal agent for bacteria ablation. GhavamiNejad et al. [65] reported a mussel-inspired electrospun nanofibers decorated with AgNPs as an antibacterial wound dressing. Jia et al. [66] presented an AgNPs immobilized onto micro-nanoporous TiO_2_ composite to prevent biomedical device-associated infections. Even though these AgNPs containing materials exhibited excellent antibacterial activity, but their uncontrolled silver release and the exceed AgNPs core could lead to potential toxicity to human body, such as spasms and gastrointestinal disorders [5, 7]. To avoid the above side effects, recently, Cao et al. [8] developed an antibacterial system by infusing low dose of Ag^+^ ions (15 µg/mL) into MoS_2_ nanosheets. This system showed outstanding antibacterial efficiency and reduced cytotoxicity. However, due to the lack of the specific bacterium-targeting, the potential toxicity of the Ag^+^ ions released from this system still could not be avoided. In contrast, our prepared antibacterial system of Ag^+^-GCS-PDA@GNRs could selectively target to the bacterial surface in acid focus of infection (pH 6.3), which would destroy bacteria without impair the normal tissues. Moreover, the dose of loaded Ag^+^ ions in our system was only 1 µg/mL, which further reduced the potential risk of silver toxicity. For the photothermal therapy (PTT), although it showed excellent bactericidal effect, it could also injury the normal tissues during the course of treatment [19]. Furthermore, the photothermal-based antibacterial agents usually killed bacteria when the temperature reached up to 55 °C, which probably burn the skin and cause irreversible damage to the host cells [14, 67]. Nevertheless, the Ag^+^-GCS-PDA@GNRs system could specially target the bacteria and efficiently eliminate bacteria at 50 °C (Figs. 3a–d and 6b, c), suggesting that application of our antibacterial system was conducive to avoid the potential skin damage caused by the high temperature. Taken together, compared with these antibacterial materials reported before, our designed Ag^+^-GCS-PDA@GNRs antibacterial system based on the synergistic chemo-phototherapy enhanced antibacterial efficacy, and mitigated the side effects of the Ag^+^ ions and the PTT alone.

### In vivo biocompatibility evaluation

To assess the potential biological application of our bactericides hydride, the in vivo biocompatibility of Ag^+^-GCS-PDA@GNRs was detected by using blood biochemistry analysis and histological examination in healthy mice. As shown in Additional file 1: Figure S6A–H, the parameters of complete blood tests in treated groups were similar as control group both at 1 day post-injection and 28 day post-injection of Ag^+^-GCS-PDA@GNRs. Moreover, no obvious difference was found in the liver function indicators of alanine amino transferase and aspartate amino transferase, as well as the kidney function markers of uric acid and creatinine among all groups (Additional file 1: Figure S6I–L). In addition, histological analysis revealed no appreciable abnormalities or damages of heart, liver, spleen, lung and kidney after intravenous injection of Ag^+^-GCS-PDA@GNRs (Additional file 1: Figure S7). Taken together, all the blood biochemistry analysis and histological examination demonstrated that the Ag^+^-GCS-PDA@GNRs had negligible short-term and long-term toxicity in vivo, which indicated its promising application in biological medicine.

The toxicity of Au and Ag on humans might be different with mice, but based on the results presented in this paper and previous studies, an initial assessment of the biosafety of Ag^+^-GCS-PDA@GNRs system on humans should be discussed. Gold nanoparticles (AuNPs) have been widely applied in the biomedical science and demonstrated to be inert and low toxic [68–70]. For example, Xia et al. [71] developed a gold nanocluster-based nanoprobe for the therapy of lung cancer, and they found that the therapeutic dose (Au: about 21.8 mg/Kg) showed no obvious in vivo toxicity to the mice. Zhang et al. also reported a chemo-photothermal theranostic platform based on polydopamine-coated gold nanorods (GNRs) for tumor ablation, and negligible toxicity was found in the major organs of mice after intravenously injected with 40 mg Au/Kg [29]. Noteworthy, the dose of Au in our system used for healing the abscess was only 4.7 mg/Kg, which was much lower than that reported in above studies. Moreover, our in vivo biochemical analysis and histological examination indicated that the nanomaterial was biocompatible (Additional file 1: Figures S6 and S7). Thus, it may well be supposed that the amount of Au in our system would be non-toxic on humans. Additionally, the in vivo therapeutic dose of Ag^+^ ions loaded in the system is about 3.9 µg/mL, which is far less than the toxic dose to human body (10 µg/mL) [72, 73], indicating that the total amount of Ag released from our system is in a safe range. Collectively, we consider that our antibacterial platform is biocompatible with humans and has a large potential in clinical therapeutic application.

## Conclusions

In summary, we developed an efficient antimicrobial hybrid for combined chemo-photothermal therapy based on PDA-coated GNRs. The PDA coating achieved high silver ions loading efficiency, and GCS functionalization (Ag^+^-GCS-PDA@GNRs). The as-designed antimicrobial hybrid could specifically target and accumulate the focal infection sites and showed an outstanding chemo-photothermal synergistic therapeutic effect on abscess, leading to sufficient bacteria eradication, wound healing acceleration, and the reduction of damage to normal tissue.

## Methods

### Materials

Hexadecyltrimethyl ammonium bromide (C_19_H_42_BrN, CTAB), potassium phosphate monobasic (KH_2_PO_4_), sodium phosphate dibasic (Na_2_HPO_4_) and ascorbic acid (C_6_H_8_O_6_, AA) were purchased from Aladdin Biotechnology Co., Ltd. (Shanghai China). *N*-(3-Dimethylaminopropyl)-*N*’-ethylcarbodiimide hydrochloride (C_8_H_17_N_3_·HCl, EDC), dopamine hydrochloride (C_8_H_11_O_2_N·HCl, DA) and N-hydroxysuccinimide (C_4_H_5_NO_3_, NHS) were obtained from J&K Co., Ltd (China). Gold (III) chloride hydrate (HAuCl_4_) was acquired from Sinopharm Group Co. Ltd. (Shanghai, China). Cy5 NHS Ester (Cy5-SE) was obtained from MedChem Express Co., Ltd. (Shanghai, China). Glycol chitosan (GCS), silver nitrate (AgNO_3_) and sodium borohydride (NaBH_4_) were obtained from Sigma-Aldrich (USA). All other chemicals were of analytical grade if not specially mentioned. The MilliQ water was used throughout the experiments.

The multidrug-resistant *Staphylococcus aureus* (MRSA, ATCC 43300) and *Escherichia coli* (*E. coli*, ATCC 25923) were obtained from the Clinical Microbiology Laboratory, Institute of Burn Research, Southwest Hospital, Third Military Medical University (TMMU, Chongqing, China). Male BALB/c mice (20–22 g) were purchased from the Experimental Animal Department of the TMMU and individually housed in plastic cages 3 days before experiments. All the animal experiments were conducted in accordance with the guidelines and the ethical standards of the Institutional Animal Care and Use Committee of the TMMU.

### Synthesis of Ag^+^-GCS-PDA@GNRs

#### Synthesis of GNRs

Gold nanorods (GNRs) were synthesized by means of seed growth method according to the previous reported [74]. Then, the mixture was centrifuged at 9000 rpm for 10 min, and washed with water for 3 times. After that, the concentrated GNR solution was obtained. The mass concentration of Au in concentrated GNR solution was examined by using the inductively coupled plasma mass spectrometry (ICP-MS, 7700X Agilent, 498.8 mg L^−1^).

#### Synthesis of PDA@GNRs

GNRs (1.5 mg) was added in 20 mL of dopamine solution (0.4 µM) buffered to pH 8.5 using 10 mM Tris–HCl buffer. The mixture was sonicated for 30 min, and then PDA@GNRs was concentrated and collected by centrifugation (6000 rpm, 30 min).

#### Synthesis of GCS-PDA@GNRs

GCS (50 mg) was firstly dissolved in 10 mL of HCl solution (0.1 mol L^−1^). Then 50 mg of GCS and 1.74 mM of NHS were slowly added into the above concentrated PDA@GNRs solution. After 2 h stirring, 5.22 mM of EDC was added in mixture under stirring for overnight. The GCS-PDA@GNRs solution was acquired through centrifugation at 6000 rpm for 30 min.

#### Synthesis of Ag^+^-GCS-PDA@GNRs

Briefly, 0.59 mM of AgNO_3_ solution was added into GCS-PDA@GNRs solution. After 6 h of stirring, the mixture was centrifuged at 7000 rpm for 10 min. Finally, the Ag^+^-GCS-PDA@GNRs solution was achieved through dialysis for 2–3 days.

#### Cy5-SE labeling

To synthesize the fluorescence labeled Ag^+^-GCS-PDA@GNRs (*f* -Ag^+^-GCS-PDA@GNRs), 2 mg of *Cy5*-*SE* was dissolved in 4 mL of dimethyl sulfoxide (DMSO). Then, the freshly made Cy5-SE solution was added into 4 mL of Ag^+^-GCS-PDA@GNRs suspension. After gently stirring for 4 h, the mixture was centrifuged at 12,000 rpm for 10 min three times to remove the free Cy5-SE. It is important to note that all of these steps need to be operated in the dark.

### Characterizations

The optical properties of Cy5-SE, GNRs, PDA@GNRs, GCS-PDA@GNRs, Ag^+^-GCS-PDA@GNRs and *f*-Ag^+^-GCS-PDA@GNRs were determined using a UV–vis-NIR spectrophotometer (UV-3600 SHIMADZU). The morphology and size of GNRs, PDA@GNRs, GCS-PDA@GNRs and Ag^+^-GCS-PDA@GNRs were examined using a transmission electron microscope (TEM-1400Plus). Fourier transform infrared spectra (FTIR) were analyzed using infrared spectrophotometer (IR Prestige-21, Shimadzu). The pH-dependent surface charge of GCS-PDA@GNRs and Ag^+^-GCS-PDA@GNRs was measured using Zetasizer Nano ZSP (Malvern, UK). The GCS-PDA@GNRs and Ag^+^-GCS-PDA@GNRs were dispersed in a range of phosphate buffer (PB, without sodium chloride) with different pH values (pH 8.0–6.0), respectively. The ratio of gold to silver was evaluated through inductively coupled plasma mass spectrometry (ICP-MS, 7700X, Agilent). To explore the loading capacity of Ag^+^ ions, an aqueous solution of Ag^+^ ions (0.2 mL) with different concentrations (25, 50, 75, 100, 125, 150 µg/mL) were added into the GCS-PDA@GNRs solution and stirred for 6 h, respectively. The amount of Ag^+^ ions was measured by using ICP-MS. To further analyze the release ability of Ag^+^ ions, Ag^+^-GCS-PDA@GNRs was suspended in PB at various pH values (6.3 and 7.4) with or without laser irradiation for a certain time (5, 10, 30 min, 1, 2, 4, 8 h), respectively. Then, the supernatant was collected with sampling (n = 3) in 4% dilute nitric acid to measure the concentration of Ag^+^ ions using ICP-MS, and the MilliQ water was processed in the same way as a control.

### Photothermal conversion efficiency in vitro

The photothermal conversion performances of GCS-PDA@GNRs or Ag^+^-GCS-PDA@GNRs were studied using a NIR laser (VLSM-808-B, Connet). For this purpose, GCS-PDA@GNRs (11.7–46.6 µg/mL) and Ag^+^-GCS-PDA@GNRs (11.7–46.6 µg/mL) were dispersed in phosphate buffer (PB, pH 6.3), respectively. After being irradiated with the laser at various power settings (0.25 and 0.5 W cm^−2^) for different times (0–11 min), the temperature and thermographic images of Ag^+^-GCS-PDA@GNRs suspension were measured by using an IR thermal camera (FLIR-E49001, Estonia). To evaluate the photostability of Ag^+^-GCS-PDA@GNRs, the absorbance intensity and thermal curves of Ag^+^-GCS-PDA@GNRs (23.3 µg/mL) were examined after repeated laser irradiation at 0.5 W cm^−2^ for 9 min (n = 5).

### In vitro specific targeting of GCS-PDA@GNRs and Ag^+^-GCS-PDA@GNRs to bacteria

To obtain the log-phase bacteria, mono-colony of MRSA and *E. coli* bacteria were grown in 4 mL of Luria–Bertani (LB) medium overnight with constant shaking at 37 °C. After being washed with sterile PBS (10 mM, pH 7.4) twice, the bacteria were diluted to a concentration of 10^6^ colony forming units (CFU)/mL in PBS (10 mM, pH 7.4 or 6.3) for the following experiments.

The interactions of GCS-PDA@GNRs or Ag^+^-GCS-PDA@GNRs (final concentration: 23.3 µg/mL) and the bacteria were assessed by using zeta potential measurement and SEM analysis. Briefly, bacteria (pH 7.4 or 6.3) were incubated alone or co-incubated with as-prepared nanomaterials (GCS-PDA@GNRs or Ag^+^-GCS-PDA@GNRs) for 30 min at 37 °C, then the mixture was washed with PBS by centrifuging at 3000 rpm for 5 min. After that, the mixture was re-suspended in 1 mL of PBS (10 mM, pH 7.4 or 6.3) and their zeta potentials were measured by a Zetasizer. To further observe the morphology of bacteria, the bacterial suspension was fixed in 4% formaldehyde overnight and then dehydrated by a series of ethanol solution (30–100%). Finally, the bacteria were dried in vacuum drying chamber and characterized by using the SEM. The amount of NP bound on the bacteria at different pH conditions (pH 7.4 & 6.3) was determined by ICP-MS.

Since keratinocytes and fibroblasts were the main composition of cutaneous tissue, HaCaT cells and 3T3 fibroblast cells were used as model cells in this experiment. Therein, to investigate whether GCS-PDA@GNRs and Ag^+^-GCS-PDA@GNRs would interact with the host tissues under physiological conditions (pH 7.4), the test nanomaterials were co-cultured with HaCaT cells or NIH3T3 fibroblasts in culture medium (pH 7.4). After 24 h, the cells were washed with PBS (10 mM, pH 7.4) twice, fixed in 4% formaldehyde and then dehydrated as described above for SEM analysis. The mass of NP bound on cells or internalized by cells after 24 h co-incubation was also detected by ICP-MS.

### In vitro antibacterial experiments and biocompatibility evaluation

To evaluate the in vitro antibacterial activity, MRSA or *E. coli* bacteria were divided into 8 groups: (1) bacteria + PBS; (2) bacteria + AgNO_3_; (3) bacteria + GCS-PDA@GNRs; (4) bacteria + Ag^+^-GCS-PDA@GNRs; (5) bacteria + PBS + NIR; (6) bacteria + AgNO_3_ + NIR; (7) bacteria + GCS-PDA@GNRs + NIR; (8) bacteria + Ag^+^-GCS-PDA@GNRs + NIR. Briefly, 800 μL of the diluted bacterial solution was mixed with 200 μL of PBS (10 mM, pH 6.3), AgNO_3_ solution (8.0 µg/mL), GCS-PDA@GNRs (116.57 µg/mL) or Ag^+^-GCS-PDA@GNRs (116.57 µg/mL) for 4 h in a 37 °C shaker with constant rotary speed. The final concentration of Ag^+^ ions (1 µg/mL) in AgNO_3_ solution was equal to that of Ag^+^-GCS-PDA@GNRs. The concentration of GCS-PDA@GNRs was equal to Ag^+^-GCS-PDA@GNRs. Then, the bacteria of NIR treated groups were further exposed to 808 nm laser (0.5 W cm^−2^) for another 7 min. Subsequently, to investigate the bactericidal activity in vitro, the bacterial viability was detected using the following standard plate counting assay [75], SEM observation and Live/Dead staining assay.

For standard plate counting assay, bacteria were uniformly spread on the agar plate and incubated at 37 °C for 18 h. The images and counting of bacterial colonies were obtained using an automatic colony counter (Supcre, Shineso, Hangzhou).

For SEM observation, the bacteria were firstly harvested by centrifugation at 3000 rpm for 5 min. Then, the bacteria were washed with PBS (10 mM, pH 7.4) for two times, and fixed in 4% formaldehyde for 12 h. Subsequently, the bacteria were dehydrated by a series of ethanol solution (30–100%). Finally, the bacteria were dried in vacuum drying chamber and sputter-coated with gold.

For Live/Dead staining assay, the bacteria were harvested by centrifugation at 3000 rpm for 5 min, and stained by the Live/Dead staining kit (Invitrogen, USA) for 15 min in dark according to the manual instructions. Then the staining bacteria were washed with PBS (10 mM, pH 7.4) twice and observed under a fluorescence microscopy (Olympus, Japan).

The dose-dependent antibacterial activity of AgNO_3_ and Ag^+^-GCS-PDA@GNRs with equal concentrations of Ag^+^ ions (0.5, 1, 2, 4, 6, 8 and 10 µg/mL) were determined by the standard plate counting assay using MRSA as the model bacterial strain. Moreover, to investigate the power-dependent bactericidal activity of NIR monotherapy, the MRSA bacterial solution co-cultured with GCS-PDA@GNRs (23.3 µg/mL) were exposed to 808 nm NIR laser with different powers (0.5, 0.75, 1.0 and 1.5 W cm^−2^) for 7 min. The number of bacterial colonies was also counted using standard plate counting assay.

The cell viability of Ag^+^-GCS-PDA@GNRs was detected by CCK8 assay. Briefly, cells (HaCaT cells or 3T3 fibroblast cells) were seeded on a 96-well plate (5000 cells/well), and incubated for 24 h in a 37 °C incubator. Subsequently, the medium was replaced with 100 µL of fresh one containing different concentrations of Ag^+^-GCS-PDA@GNRs (11.7, 23.3 and 46.6 µg/mL) and irradiated with NIR (0.5 W cm^−2^) for 7 min. After 24 h co-incubation, the medium was refreshed with 100 µL of one containing 10 µL CCK8 solution (Dojindo, Japan), and cultured at 37 °C for another 2 h. Finally, the optical density value of the medium was obtained using a microplate reader (Thermo Varioskan Flash, USA) recorded at 450 nm.

### Hemolysis assay of Ag^+^-GCS-PDA@GNRs

The hemolysis assay was performed using fresh human blood obtained from the Southwest Hospital, Chongqing, China. Briefly, the whole blood was centrifuged at 1500 rpm for 15 min to obtain erythrocytes. After washed with saline twice, 3 mL of the erythrocytes were added into 11 mL of saline to prepare the stock dispersion. Then 100 µL of stock dispersion were mixed with 1 mL of different concentrations of Ag^+^-GCS-PDA@GNRs (11.7, 23.3 and 46.6 µg/mL), MilliQ water or saline. After incubated at 37 °C for 3 h, the mixture solution was centrifuged at 10,000 rpm for 15 min, and then the absorbance of supernatant at 540 nm was measured using a microplate reader (Thermo Varioskan Flash, USA). Here, the MilliQ water was served as positive control group, and the saline was served as negative control group. The percentage of hemolysis was calculated with the following formula:

$$ {\text{Hemolysis ratio}}\;\left( \% \right)\, = \,{{\left( {A_{G} \, - \,A_{N} } \right)} \mathord{\left/ {\vphantom {{\left( {A_{G} \, - \,A_{N} } \right)} {\left( {A_{P} \, - \,A_{N} } \right)\,}}} \right. \kern-0pt} {\left( {A_{P} \, - \,A_{N} } \right)\,}} \times \, 100\% $$

where *A*_*G*_ is the absorbance of Ag^+^-GCS-PDA@GNRs group, *A*_*N*_ is the absorbance of saline group, and *A*_*P*_ is the absorbance of water group.

### In vivo biodistribution and thermographic images

Male Balb/c mice (∼ 20 g) were anesthetized with an intraperitoneal injection of 1% pentobarbital, and the dorsal surface of mice was shaved and cleaned with 70% alcohol. Subsequently, 50 µL of MRSA suspension (1 × 10^9^ CFU/mL) was injected subcutaneously into the right flank, while 50 µL of PBS (pH 7.4) was injected subcutaneously into the left flank as a control. After 24 h, 100 µL of Ag^+^-GCS-PDA@GNRs was intravenously injected at a dose of 4.7 mg/Kg. The mice were then anesthetized with 1% pentobarbital, and the near infrared fluorescence (NIRF) images were taken using an IVIS Lumina imaging system at determined time points (0, 2, 4, 6 and 24 h). Next, at 24 h post-injection, the mice were sacrificed and the various organs including heart, liver, spleen, lung, kidney and abscess were harvested, and these tissues were also imaged by an IVIS Lumina imaging system. Finally, all the tissues were each homogenized and dissolved in aqua regia, and the mass of Au and Ag was detected using ICP-MS to obtain the percentage of injected dose per gram of tissue (% ID/g) [76]. In addition, to further confirm the in vivo targeting capacity of Ag^+^-GCS-PDA@GNRs, each injected side of the back of test mouse was separately irradiated with an 808 nm NIR laser (0.8 W cm^−2^, 6 min) at the pre-determined time intervals (0, 2, 4, 6 and 24 h), and the thermographic images were taken by an IR thermal camera (FLIR-E49001, Estonia).

Furthermore, to evaluate the ability of GCS-PDA@GNRs and Ag^+^-GCS-PDA@GNRs to produce in vivo hyperthermia at abscess, infected mice were intravenously injected with 100 µL of PBS (10 mM, pH 6.3), GCS-PDA@GNRs (4.7 mg/Kg) or Ag^+^-GCS-PDA@GNRs 4.7 mg/Kg), respectively. After 6 h, the abscess of each mice was irradiated under an 808 nm NIR laser (0.8 W cm^−2^) for 0–8 min, and the temperature was monitored using an IR thermal camera (FLIR-E49001, Estonia).

### In vivo antibacterial efficiency of Ag^+^-GCS-PDA@GNRs

To investigate the in vivo antibacterial activity of Ag^+^-GCS-PDA@GNRs, a murine subcutaneous abscess model was performed. Firstly, the BALB/c mice were anesthetized with an intraperitoneal injection of 1% pentobarbital, and the dorsal surface of mice were shaved and cleaned with 70% alcohol. Subsequently, 50 *µ*L of MRSA suspension (1 × 10^9^ CFU/mL) was injected subcutaneously into both the left and right sides of skin. Twenty-four hours later, an obvious subcutaneous abscess was arisen in each side of the dorsal surface. Then, the mice were randomly divided into seven groups (5 mice per group): (1) Control group (PBS injection), (2) PBS + NIR group, (3) AgNO_3_ group, (4) GCS-PDA@GNRs group, (5) Ag^+^-GCS-PDA@GNRs group, (6) GCS-PDA@GNRs + NIR group, and (7) Ag^+^-GCS-PDA@GNRs + NIR group. Briefly, 100 *µ*L of PBS (10 mM, pH 6.3), AgNO_3_ (0.3 mg/Kg), GCS-PDA@GNRs (4.7 mg/Kg) or Ag^+^-GCS-PDA@GNRs (4.7 mg/Kg) were intravenously injected into mice of each group, respectively. Six hours later, the mice under anesthesia (for NIR treated groups) were exposed to 808 nm NIR laser for 6 min (0.8 W cm^−2^). After 9 days, the abscess was photographed and the infected tissues were harvested for further histological analysis. In addition, to evaluate the bactericidal activity in vivo, the infected tissues (10 × 10 mm) were immersed in 5 mL of physiological saline solution and homogenized. Then, the number of bacteria was counted using standard plate counting assay.

### In vivo biocompatibility evaluation

The mice were injected with 100 µL of PBS (10 mM, pH 7.4) or Ag^+^-GCS-PDA@GNRs solution at a dose twofold that of the treatment dose (9.4 mg/Kg) through the tail vein. These mice were sacrificed at 1 or 28 days post-injection, then the blood samples were collected and the blood biochemistry analysis was performed. Additionally, the major organs including heart, liver, spleen, lung and kidney were also harvested for histological analysis at determined time points. The mice injected with PBS were severed as control and four mice were used for each group.

### Statistical analysis

The experimental data are expressed as mean ± standard deviation (SD), and the significant difference between groups was analyzed using unpaired t test (for two groups) and one-way analysis of variance (ANOVA) (for more than two groups) in the Origin software. The statistical significance was set as *p *< 0.05 (“*”) and *p *< 0.01 (“**”).

## Additional file

**Additional file 1: Figure S1.** FTIR spectra of GNRs, PDA, GCS, PDA@GNRs, GCS-PDA@GNRs and Ag+-GCS-PDA@GNRs. **Figure S2**. The zeta potential of GNRs, PDA@GNRs and GCS-PDA@GNRs. **Figure S3**. UV-vis-NIR and fluorescence spectra of CY5-SE, Ag+-GCS-PDA@GNRs and f-Ag+-GCS-PDA@GNRs. **Figure S4**. Temperature evolution profile of PB buffer (pH = 6.3) and Ag+-GCS-PDA@GNRs suspensions with different concentrations in PB upon NIR laser (808 nm, 0.25 W cm^−2^) irradiation. **Figure S5**. The blood compatibility of different concentrations of Ag+-GCS-PDA@GNRs. **Figure S6**. The parameters of complete blood tests, liver and kidney function markers of the mice after intravenously injection of Ag+-GCS-PDA@GNRs at 1 day post-injection and 28 days post-injection. **Figure S7**. The cytotoxic effect caused by Ag+-GCS-PDA@GNRs on major organs of mice after intravenous injection of 1 day and 28 days, respectively.
