# Supplementary material for: An efficient antimicrobial depot for infectious site-targeted chemo-photothermal therapy
Source: J Nanobiotechnology. 2018 Mar 16;16:23. doi: 10.1186/s12951-018-0348-z (PMC5857115; doi:10.1186/s12951-018-0348-z)
Supplement: Supplementary file 1 — Additional file 1: Figure S1. FTIR spectra of GNRs, PDA, GCS, PDA@GNRs, GCS-PDA@GNRs and Ag+-GCS-PDA@GNRs. Figure S2. The zeta potential of GNRs, PDA@GNRs and GCS-PDA@GNRs. Figure S3. UV-vis-NIR and fluorescence spectra of CY5-SE, Ag+-GCS-PDA@GNRs and f-Ag+-GCS-PDA@GNRs. Figure S4. Temperature evolution profile of PB buffer (pH = 6.3) and Ag+-GCS-PDA@GNRs suspensions with different concentrations in PB upon NIR laser (808 nm, 0.25 W cm−2) irradiation. Figure S5. The blood compatibility of different concentrations of Ag+-GCS-PDA@GNRs. Figure S6. The parameters of complete blood tests, liver and kidney function markers of the mice after intravenously injection of Ag+-GCS-PDA@GNRs at 1 day post-injection and 28 days post-injection. Figure S7. The cytotoxic effect caused by Ag+-GCS-PDA@GNRs on major organs of mice after intravenous injection of 1 day and 28 days, respectively. [file 12951_2018_348_MOESM1_ESM.doc]

Additional file 1

**An Efficient Antimicrobial Depot for Infectious Site-Targeted Chemo-Photothermal Therapy**

Menglong Liu^a#^, Danfeng He^a#^, Tao Yang^a^, Wei Liu^a^, Li Mao^b^, Yang Zhu^d^, Jun Wu^a,c*^, Gaoxing Luo^a*^, Jun Deng^a*^

1. Institute of Burn Research, Southwest Hospital, State Key Lab of Trauma, Burn and Combined Injury, Third Military Medical University (Army Medical University), Chongqing 400038, China.
2. Department of Laboratory Medicine, Southwest Hospital, Third Military Medical University (Army Medical University), Chongqing 400038, PR China
3. Department of Burns, The First Affiliated Hospital, SunYat-Sen University, Guangzhou 510080, China.
4. Departments of Bioengineering and Materials Science and Engineering, University of California, Berkeley, CA 94720,

^#^ Co-first authors.

* Corresponding authors.

E-mail addresses: editorinchief@burninchina.com (J. Wu), [logxw@yahoo.com](mailto:logxw@yahoo.com) (G. Luo), [djun.123@163.com](mailto:djun.123@163.com) (J. Deng)

**Figure S1.** FTIR spectra of (A) GNRs, PDA and GCS, and (B) PDA@GNRs, GCS-PDA@GNRs and Ag^+^-GCS-PDA@GNRs.

**Figure S2.** The zeta potential of (A) GNRs and PDA@GNRs in water, and (B) GCS-PDA@GNRs in PB with different pH values, respectively.

**Figure S3.** (A) UV-vis-NIR and (B) fluorescence spectra of CY5-SE, Ag^+^-GCS-PDA@GNRs and *f*-Ag^+^-GCS-PDA@GNRs, respectively.


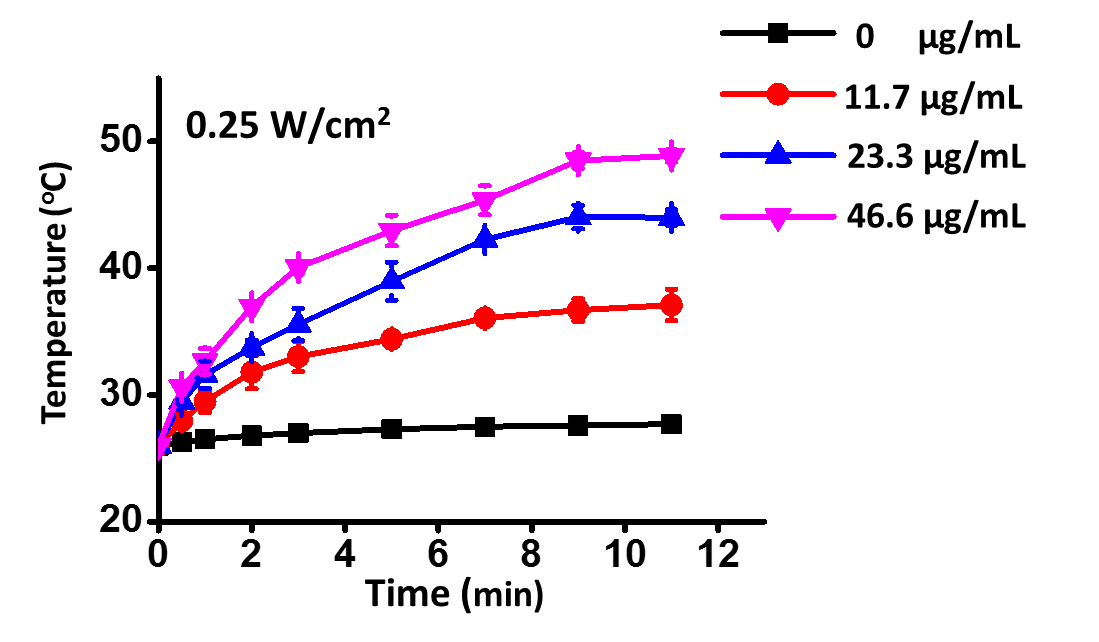


**Figure S4.** Temperature evolution profile of PB buffer (pH=6.3) and Ag^+^-GCS-PDA@GNRs suspensions with different concentrations in PB upon NIR laser (808 nm, 0.25 W·cm^-2^) irradiation.


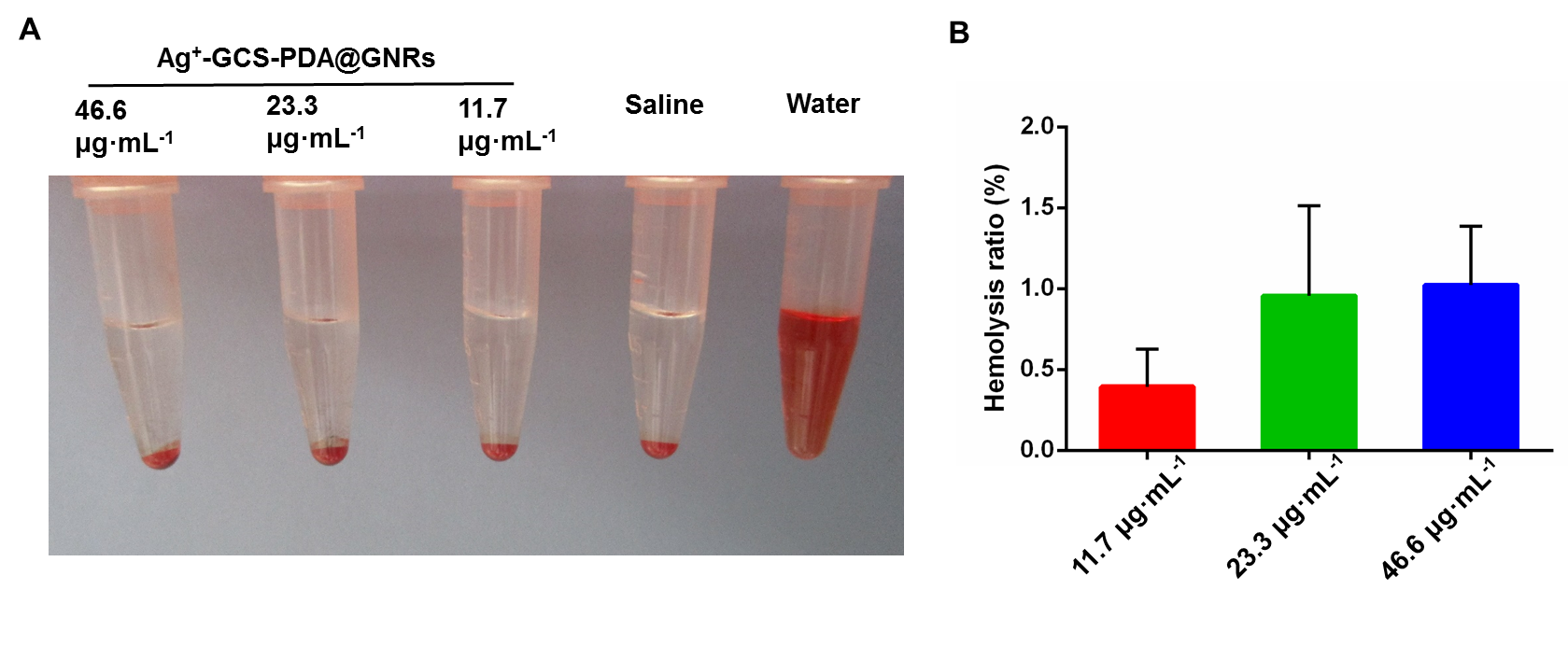


**Figure S5.** (A) Photographs of human blood incubated with different doses of Ag^+^-GCS-PDA@GNRs and (B) corresponding hemolysis ratio of different concentrations of Ag^+^-GCS-PDA@GNRs.

**Figure S6.** The parameters of complete blood tests (A–H) of mice after intravenously injection of Ag^+^-GCS-PDA@GNRs at a dose of 9.4 mg/Kg at 1 day post-injection and 28 days post-injection. The data of liver (I, J) and kidney (K, L) function markers of the test mice at 1 day and 28 days post-injection, respectively.

**Figure S7.** Pathological study of the cytotoxic effect caused by Ag^+^-GCS-PDA@GNRs (9.4 mg/Kg) on major organs (heart, liver, spleen, lung and kidney) after intravenous injection of 1 day and 28 days, respectively. Scale bars: 100 μm.
